# A Multi‐Targeting Chimeric Antigen Receptor‐T Cell Platform to Overcome Antigen Heterogeneity in the Treatment of Non‐Small Cell Lung Cancer

**DOI:** 10.1002/advs.77992

**Published:** 2026-09-27

**Authors:** Lina Wang, Yanlei Zhang, Shuixiu Peng, Chenchen Hou, Huaqi Guo, Tianyu Zhou, Alex H. Chang, Lifeng Yan, Weining Xiong

**Affiliations:** ^1^ Department of Pulmonary and Critical Care Medicine Shanghai Key Laboratory of Tissue Engineering Shanghai Ninth People's Hospital Shanghai Jiaotong University School of Medicine Shanghai China; ^2^ Institute of Respiratory Diseases Shanghai Jiao Tong University School of Medicine Shanghai China; ^3^ YaKe Center for Cell Engineering and Therapeutics Shanghai China; ^4^ Engineering Research Center of Gene Technology Ministry of Education Institute of Genetics School of Life Sciences Fudan University Shanghai China

**Keywords:** antigen heterogeneity, bicephali, CAR‐T cells, CD276, NKG2D ligands, NSCLC

## Abstract

Chimeric antigen receptor (CAR)‐T cell therapy for solid tumors is limited by antigen heterogeneity and T cell exhaustion. To address these limitations, we develop a novel multi‐targeting “Bicephali” CAR‐T platform featuring a dual‐transmembrane protein with two distinct extracellular antigen‐binding domains and a shared intracellular 4‐1BB co‐stimulatory/CD3ζ signaling domain. CD276 and NKG2D ligands (NKG2DLs) show high and heterogeneous expression in non‐small cell lung cancer (NSCLC) and are undetectable in normal tissues. Bicephali CAR‐T cells targeting CD276 and NKG2DLs demonstrate superior tumoricidal activity against NSCLC than conventional BB002 CAR‐T cells in vitro and in vivo, together with improved immunological synapse formation and mitochondrial metabolic fitness. In homogeneous NSCLC models co‐expressing CD276 and NKG2DLs, Bicephali CAR‐T cells achieve prolonged survival outcomes compared to monospecific CAR‐T cells. In antigenically heterogeneous NSCLC, Bicephali CAR‐T cells more consistently control tumors and prolong survival, whereas monospecific CAR‐T cells fail to eliminate tumors following antigen loss. Mechanistically, improved mitochondrial fitness and antioxidant capacity in Bicephali CAR‐T cells are associated with sustained T cell function, preserved stem‐like differentiation, and durable effector responses. These findings support a multi‐targeting CAR‐T approach to address antigen heterogeneity in NSCLC and potentially other solid tumors.

## Graphical abstract

A dual‐transmembrane Bicephali CAR‐T platform simultaneously targets CD276 and NKG2D ligands to address antigen heterogeneity in non‐small cell lung cancer. By improving immune synapse formation, mitochondrial fitness, antioxidant capacity, and stem‐like persistence, this design enhances durable tumor control and provides a versatile strategy to overcome antigen escape in solid tumors.

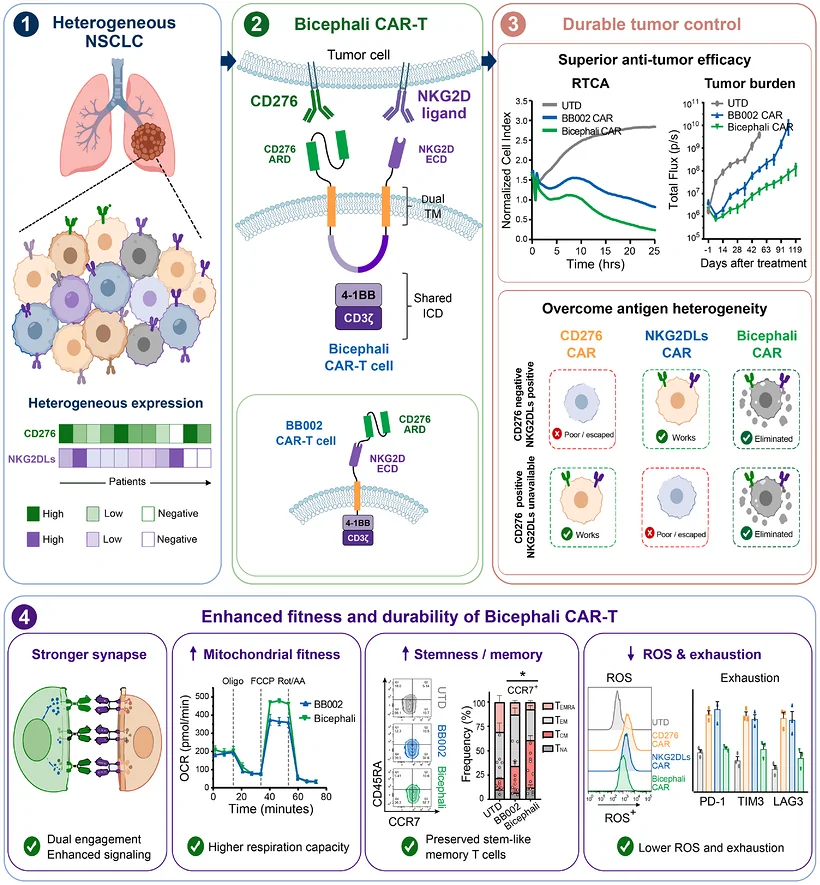

## 1 Introduction

Over the past decades, chimeric antigen receptor (CAR)‐T cell therapy has emerged as an attractive cancer immunotherapeutic strategy [1, 2, 3]. Genetic modification of T cells to express CARs enables antigen‐specific tumor recognition and anti‐tumor capability [4]. Since the US Food and Drug Administration (FDA) approved tisagenlecleucel (Kymriah) and axicabtagene ciloleucel (Yescarta) for the treatment of relapsed/refractory acute lymphoblastic leukemia and large B‐cell lymphoma in 2017 [5, 6], CAR‐T cell therapy has achieved remarkable success in hematological malignancies. However, the achievements of CAR‐T cells have not been recapitulated in solid tumors [7].

The remarkable efficacy of CAR‐T therapy in hematological malignancies is primarily attributed to relatively homogeneous antigen expression and a less complex tumor microenvironment (TME). In contrast, solid tumors exhibit heterogeneous antigen profiles and a highly immunosuppressive TME [8]. Under selective pressure, tumor cells can evade immune recognition through antigen loss or downregulation, a process termed antigen escape [9]. This mechanism can reduce CAR‐T cell efficacy and contribute to treatment failure. To address antigen escape, multi‐targeting CAR‐T cells capable of recognizing at least two distinct antigens are under active development [10]. In this case, CAR‐T cell activation can be triggered upon engagement of either target antigen, thereby mitigating antigen escape. Current dual‐targeting strategies commonly incorporate two antigen‐binding domains into a single transmembrane CAR backbone with shared moiety of co‐stimulation and CD3ζ signaling [10, 11]. Although this arrangement may improve expression efficiency and consistency of CARs, the fusion of two single‐chain variable fragments (scFvs) within a unified architecture can introduce steric hindrance and spatial constraints, thereby compromising antigen sensitivity and downstream signaling [12]. Therefore, it remains a key challenge in bispecific and multi‐targeting CAR design in determining the optimal linkage and spatial arrangement of antigen‐recognition domains to ensure effective binding while minimizing steric interference [13]. Moreover, conventional dual‐targeting CARs may exhibit diminished recognition of tumor cells expressing only one target due to lower target‐specific affinity or impaired signaling [11]. These limitations support the development of alternative multi‐targeting CAR‐T strategies to improve therapeutic outcomes against solid tumors.

CD276 (also known as B7‐H3) and natural killer group 2 member D ligands (NKG2DLs) have emerged as promising targets for CAR‐T cell‐based immunotherapy against solid tumors because of their preferential expression in tumors [14, 15, 16, 17]. CD276, a type I transmembrane protein belonging to the B7 family of immune checkpoint proteins, exerts negative immunoregulatory effects [18]. It is overexpressed in a variety of solid tumors and is involved in tumorigenesis and malignant behaviors [19]. NKG2D is an activating receptor naturally expressed on NK cells, NKT cells, and subsets of γδ T cells [20], and binds eight ligands, including UL‐16 binding proteins 1–6 (ULBP1‐6), and MHC class I chain‐related molecules A and B (MICA and MICB) [21]. These ligands have limited expression in benign cells and are upregulated in several malignancies [22, 23]. Preclinical and clinical studies have evaluated CAR‐T cells targeting CD276 or NKG2DLs in a range of malignant tumors, including ovarian cancer, hepatocellular carcinoma, and gastrointestinal cancers [24, 25, 26, 27].

Lung cancer remains a leading cause of cancer incidence and mortality worldwide [28]. In 2022, approximately 2.5 million new cases and over 1.8 million deaths were attributed to lung cancer, accounting for nearly 1/8 of cancer diagnoses and 1/5 of cancer‐related deaths [28]. Non‐small cell lung cancer (NSCLC) constitutes approximately 85% of all cases, encompassing lung adenocarcinoma (LUAD), lung squamous cell carcinoma (LUSC), and large cell carcinoma [29]. NSCLC is characterized by substantial biological and histopathological heterogeneity, which complicates the development of broadly effective targeted therapies [30, 31].

Here, we developed a novel multi‐targeting CAR‐T platform termed “Bicephali”. This multi‐targeting CAR design incorporates a dual‐transmembrane architecture with two distinct extracellular antigen‐binding domains and shared intracellular co‐stimulatory and CD3ζ signaling domains. Using this platform, we engineered multi‐targeting CAR‐T cells capable of recognizing CD276 and all eight NKG2DLs. Our studies revealed that Bicephali CAR‐T cells were capable of overcoming antigen heterogeneity and exhibited enhanced anti‐tumor efficacy. Mechanistically, Bicephali CAR‐T cells demonstrated improved mitochondrial fitness and enhanced antioxidant capacity that underpins sustained T cell functionality, promoting stem‐like differentiation and durable effector responses. These findings support Bicephali as a multi‐targeting CAR design paradigm for NSCLC and other solid tumors with heterogeneous antigen expression.

## 2 Results

### 2.1 CD276 and NKG2DLs are Highly and Heterogeneously Expressed in Human NSCLC Tissues and Cell Lines

**FIGURE 1:**
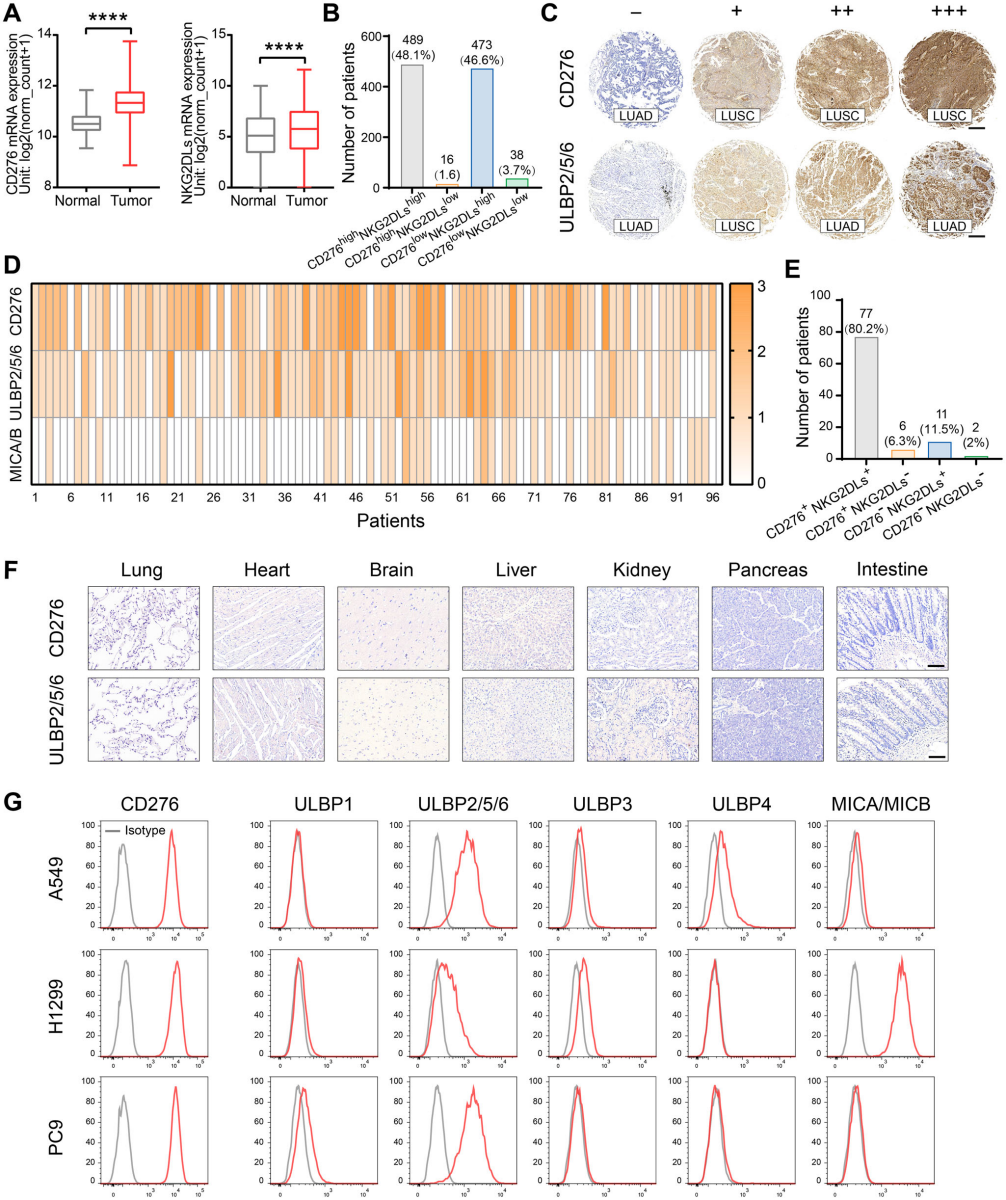
CD276 and NKG2D ligands (NKG2DLs) are highly and heterogeneously expressed in NSCLC. (A) Box plots showing mRNA expression of CD276 (left) and NKG2DLs (right) in human normal (n  =  110) and NSCLC (n  =  1016) samples based on TCGA. The whiskers indicate minimum to maximum values. An unpaired, two‐tailed Student's *t*‐test was used to calculate the significance of the difference between the 2 groups. ****P < 0.0001. (B) Classification of NSCLC samples from the TCGA cohort (n = 1016) based on CD276 and NKG2DLs expression status. Patients were stratified into four subgroups using median cutoffs: CD276^high^NKG2DLs^high^, CD276^high^NKG2DLs^low^, CD276^low^NKG2DLs^high^, and CD276^low^NKG2DLs^low^. (C) Representative IHC images showing negative (–), weak positive (+), moderate positive (++), and strong positive (+++) staining of CD276 (upper panel) and NKG2DLs (ULBP2/5/6) (lower panel) in NSCLC tumor tissues from independent patients (n = 96). The histological subtype of each representative specimen, classified as lung adenocarcinoma (LUAD) or lung squamous cell carcinoma (LUSC), is indicated in the images. Scale bar  =  300 µm. (D) Heatmap of qualitative IHC scores for CD276 and NKG2DLs (ULBP2/5/6 and MICA/MICB) in 96 NSCLC specimens from the tissue microarrays. A score of 0 indicates negative, and 1–3 signifies weak, moderate, or strong positive, respectively. (E) Validation of CD276/NKG2DLs co‐expression patterns in an independent NSCLC cohort (n = 96) using tissue microarrays. Subgroup frequencies were determined by immunohistochemistry (IHC) scoring (negative = 0; positive = 1–3). (F) Representative IHC images of CD276 (upper panel) and NKG2DLs (ULBP2/5/6) (lower panel) expression in normal human tissues including lung, heart, brain, liver, kidney, pancreas, and intestine (n = 4). Scale bar = 100 µm. (G) Detection of CD276 and NKG2DLs expression by flow cytometry in human NSCLC cell lines.

To characterize CD276 and NKG2DLs expression in NSCLC, we analyzed the mRNA transcriptome data from the TCGA database, comprising 1016 NSCLC tumor tissues (514 LUAD and 502 LUSC) and 110 normal lung tissues. Both CD276 and NKG2DLs were expressed at significantly higher levels in NSCLC tissues than that in normal lung tissues (Figure 1A and Figure S1A). We then stratified CD276 and NKG2DLs expression as high or low using median expression values to visualize co‐expression patterns within individual primary samples (Figure 1B). Concurrent high expression of CD276 and NKG2DLs was observed in 48.1% of patients, whereas 48.2% showed discordant expression (CD276^high^NKG2DLs^low^ or CD276^low^NKG2DLs^high^), demonstrating substantial heterogeneity in antigen co‐expression. We further assessed the expression of CD276 and NKG2DLs (ULBP2/5/6 and MICA/MICB) by immunohistochemistry (IHC) in clinical NSCLC specimens and multiple normal organ tissues. IHC revealed high but heterogeneous cell membrane expression of CD276 and NKG2DLs in NSCLC tissues (Figure 1C,D), among which 80.2% of cases exhibited concurrent expression of both antigens, while monoantigenic expression (17.8%) provided additional evidence for immunophenotypic heterogeneity (Figure 1E). Moreover, no CD276 or NKG2DLs staining was detected in the normal tissues examined, including the lung, heart, brain, liver, kidney, pancreas, and intestine (Figure 1F). Flow cytometry of human NSCLC cell lines further showed uniformly high CD276 expression, whereas surface NKG2DLs expression varied among different cell lines, with ULBP2/5/6 consistently prominent (Figure 1G and Figure S1B).

Together, these data show that CD276 and NKG2DLs are frequently overexpressed in NSCLC, while being undetectable in vital normal organs. However, their heterogeneous expression across tumors provides a rationale for simultaneously targeting both antigen classes to reduce the risk of antigen escape and improve therapeutic efficacy.

### 2.2 Characterization and Optimization of CD276/NKG2DLs Multi‐Targeting CAR‐T Cells

**FIGURE 2:**
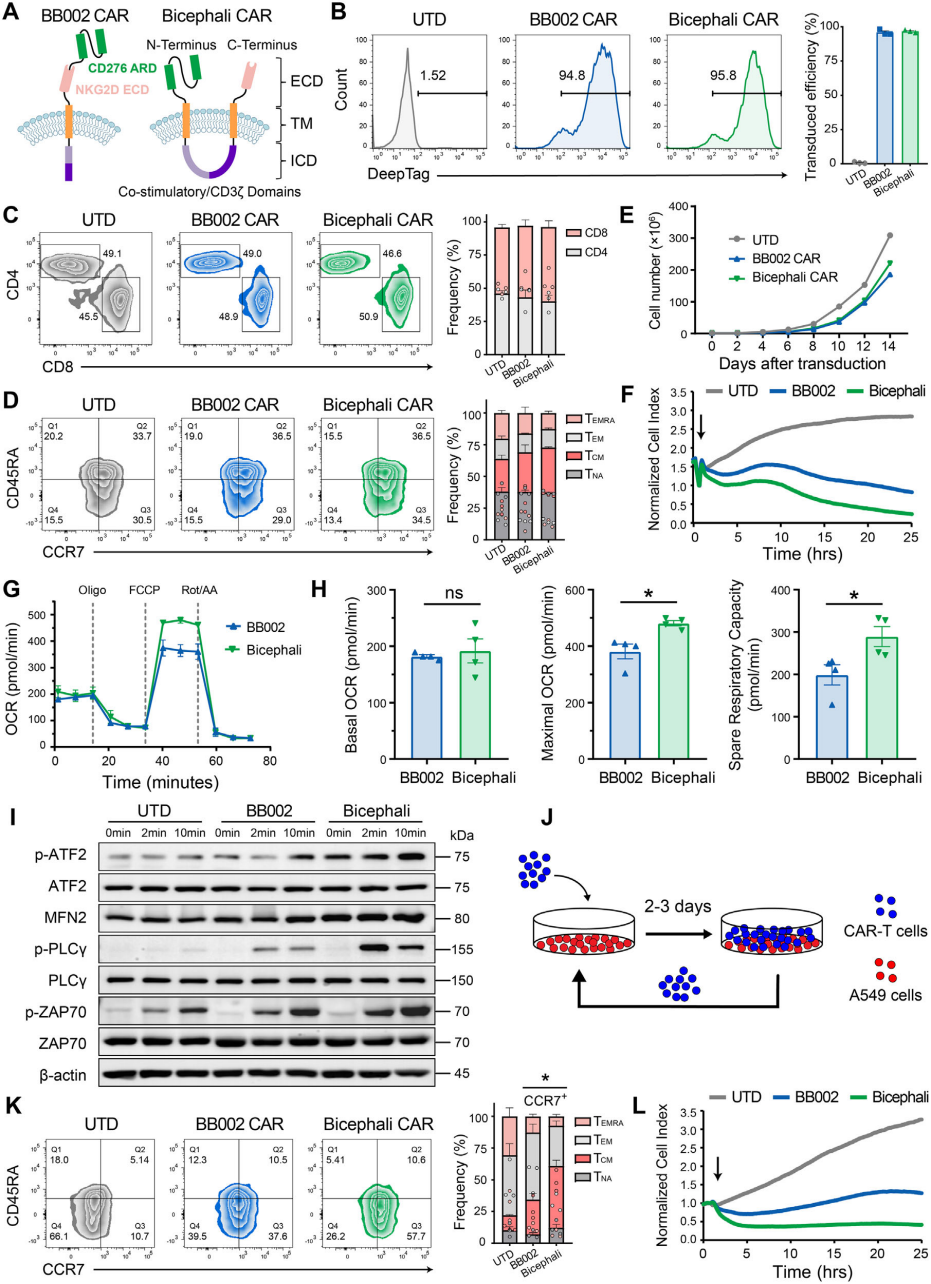
Bicephali CAR‐T cells outperform conventional CAR construct through enhanced mitochondrial fitness. (A) Schematic diagram of conventional BB002 (left) and Bicephali (right) CD276/NKG2DLs multi‐targeting CAR constructs. ARD, antigen recognition domain; ECD, extracellular domain; TM, transmembrane domain; ICD, intracellular domain. (B) Transduction efficiency detected by flow cytometry and reported by the DeepTag. Data are presented as mean ± SEM of untransduced T (UTD) or CAR‐T cells generated from three independent donors. (C) Representative plots (left) and mean frequencies ± SEM (right) of CD4^+^/CD8^+^ ratios for UTD and CAR‐T cells generated from three independent donors. (D) Representative plots (left) and mean frequencies ± SEM (right) of naïve/stem cell‐like memory T cells (T_NA_, CD45RA^+^/CCR7^+^), central memory T cells (T_CM_, CD45RA^−^/CCR7^+^), effector memory T cells (T_EM_, CD45RA^−^/CCR7^−^), and terminally differentiated effector memory T cells (T_EMRA_, CD45RA^+^/CCR7^−^) in UTD and CAR‐T cells generated from three independent donors. (E) Expansion curves of UTD and CAR‐T cells after being cultured in vitro for 14 days. Data are presented as mean ± SEM of UTD or CAR‐T cells generated from three independent donors. (F) xCELLigence real‐time cell analysis (RTCA) was used to monitor the cytolysis of CD276 and NKG2DLs multi‐targeting CAR‐T cells against the human NSCLC tumor cell line A549. Representative cytotoxicity data from three independent experiments using T cells from three healthy donors are shown. (G) Mitochondrial fitness of CAR‐T cells was assessed using Seahorse analysis 24 h post‐stimulation with A549 tumor cells. The plot shows the oxygen consumption rate (OCR) before and after treatment with oligomycin (Oligo), carbonyl cyanide 4‐(trifluoromethoxy) phenylhydrazone (FCCP), and rotenone and antimycin (Rot/AA). Data are presented as mean ± SEM of four technical replicates from one representative donor. (H) Basal OCR, maximal OCR and spare respiratory capacity (SRC) from one representative donor. Differences between groups were assessed using an unpaired, two‐tailed Student's *t*‐test. *p < 0.05. (I) UTD and CAR‐T cells were stimulated with A549 cells and lysed at the indicated time points. Western blotting was performed to detect relevant indicators. The corresponding molecular weights are indicated in kDa. Data are representative of three independent experiments. (J) Overview of the in vitro continuous antigen exposure (CAE) model of CAR‐T cells co‐cultured with A549 cells. (K) Representative plots (left) and mean frequencies ± SEM (right) of naïve/stem cell‐like memory T cells (T_NA_, CD45RA^+^/CCR7^+^), central memory T cells (T_CM_, CD45RA^−^/CCR7^+^), effector memory T cells (T_EM_, CD45RA^−^/CCR7^−^), and terminally differentiated effector memory T cells (T_EMRA_, CD45RA^+^/CCR7^−^) in UTD and CAR‐T cells generated from three independent donors after five cycles of CAE. Differences between groups were assessed using an unpaired, two‐tailed Student's *t*‐test. *p < 0.05. (L) Normalized cell index reflecting the cytotoxicity of UTD and two multi‐targeting CAR‐T cell products against A549 cells after five cycles of CAE. Representative cytotoxicity data from three independent experiments using T cells from three healthy donors are shown.

Given the frequent and heterogeneous expression of CD276 and NKG2DLs in NSCLC, we engineered multi‐targeting CAR‐T cells that simultaneously recognize both antigens to enhance anti‐tumor efficacy. Conventional dual‐target CAR architectures can be constrained by suboptimal spatial arrangement of antigen‐binding domains and steric interference between linked scFvs. We therefore developed the “Bicephali” CAR, a dual‐transmembrane architecture in which two independent antigen‐recognition and transmembrane domains converge on shared intracellular 4‐1BB co‐stimulatory and CD3ζ signaling domains (Figure 2A). For comparison, we constructed a conventional single‐transmembrane construct (“BB002”) containing both extracellular antigen‐targeting domains and the same intracellular signaling motifs. Both CAR constructs incorporated a BCMA‐derived detection tag (DeepTag) and exhibited efficient, comparable surface expression on day 6 after lentiviral transduction (Figure 2B). CAR expression remained stable through day 14 after transduction (Figure S2A,B). Staining of the same CAR‐T cell batch with an anti‐NKG2D monoclonal antibody (mAb) on day 6 yielded results consistent with DeepTag‐based detection (Figure S2C), providing an independent confirmation of CAR expression. The two CAR‐T cell products further showed comparable CD4^+^/CD8^+^ T cell composition (Figure 2C), memory differentiation (Figure 2D), and proliferative capacity during 14 days of expansion (Figure 2E).

Given that the spatial arrangement of the two targeting modules could influence CAR expression and function, we next tested alternative orientations of the CD276 antigen recognition domain (ARD) and NKG2D extracellular domain (ECD). An NKG2DLs‐CD276 BB002 CAR construct, with the NKG2D ECD positioned upstream of the CD276 ARD, showed inferior transduction efficiency (Figure S3A) and cytotoxic activity (Figure S3B–G) than the CD276‐NKG2DLs BB002 configuration, indicating that reversal of the two targeting modules did not improve CAR‐T cell generation or anti‐tumor function. We then evaluated NKG2D ECD positioning in the context of NKG2D‐based CAR design by comparing a construct with the NKG2D ECD at the N‐terminus (NKG2DLs‐BB002 CAR) with one carrying the NKG2D ECD at the C‐terminus (NKG2DLs‐Bicephali CAR). The C‐terminal NKG2DLs‐Bicephali CAR showed higher CAR expression and greater target‐cell cytotoxicity (Figure S3H–J). This orientation is consistent with the native topology of NKG2D, a type II transmembrane receptor whose extracellular ligand‐binding domain is located at the C‐terminus. We therefore selected the CD276‐NKG2DLs configuration for subsequent experiments.

### 2.3 Bicephali Multi‐Targeting CAR‐T Outperforms Conventional BB002 CAR Construct Through Improved Immunological Synapse Assembly and Mitochondrial Fitness

After establishing the optimal spatial arrangement of the CD276 and NKG2DLs targeting modules, we compared the cytotoxicity of Bicephali and BB002 multi‐targeting CAR‐T cells. Real‐Time Cell Analysis (RTCA) demonstrated that the Bicephali CAR‐T cells mediated more rapid and complete eradication of NSCLC tumor cell lines than conventional BB002 CAR‐T cells did (Figure 2F and Figure S4A,B), indicating that Bicephali CAR‐T cells have superior anti‐tumor efficacy in vitro. Consistently, Bicephali CAR‐T cells secreted higher levels of IL‐2, IFN‐γ, TNF‐α, granzyme B, and perforin upon stimulation with NSCLC cell lines (Figure S4C–G) and showed greater antigen‐dependent proliferation than BB002 CAR‐T cells, as assessed by CFSE dilution assays (Figure S4H–J).

To determine whether the functional advantage of Bicephali CAR‐T cells was associated with differences in target‐cell engagement, we compared immunological synapse formation between CD276/NKG2DLs Bicephali and conventional BB002 CAR‐T cells [32]. Bicephali CAR‐T cells formed larger and more organized synaptic interfaces, with greater F‐actin accumulation and a shorter distance between the microtubule organizing center (MTOC) and the synapse than BB002 CAR‐T cells (Figure S5). These findings indicate more efficient synapse assembly and intracellular polarization in Bicephali CAR‐T cells and are consistent with improved target‐cell engagement.

Given the established role of mitochondrial metabolism in T cell function [33], we next examined whether the improved synapse phenotype of Bicephali CAR‐T cells was accompanied by differences in metabolic fitness. Seahorse analysis showed higher maximal respiratory capacity and spare respiratory capacity in Bicephali than in conventional BB002 CAR‐T cells (Figure 2G,H), indicating improved mitochondrial fitness. Western blot analysis further showed increased phosphorylation of activating transcription factor 2 (ATF2), which is involved in metabolic adaptation, together with higher expression of mitofusin‐2 (MFN2), a critical regulator of mitochondrial fusion and bioenergetics (Figure 2I) [34]. Notably, the improved synapse organization observed in Bicephali CAR‐T cells was accompanied by stronger proximal activation signaling. Specifically, Bicephali CAR‐T cells showed increased phosphorylation of phospholipase C gamma (PLCγ) and Zeta‐chain‐associated protein 70 (ZAP70) following tumor‐cell stimulation (Figure 2I), suggesting that enhanced synapse assembly is linked to more efficient signal initiation and propagation. Together, these results support a coordinated model in which the Bicephali architecture promotes more effective immune synapse formation, thereby facilitating stronger proximal signaling and downstream metabolic remodeling.

To assess whether these advantages translated into improved durability under chronic antigen pressure, we employed a continuous antigen exposure (CAE) in vitro model in which CAR‐T cells underwent five serial stimulations with A549 cells (Figure 2J). After repeated challenge, Bicephali CAR‐T cells retained a higher proportion of naïve and central memory T cells than conventional BB002 CAR‐T cells did (Figure 2K). Moreover, only Bicephali CAR‐T cells maintained significant tumor cytotoxicity after five rounds of co‐culture (Figure 2L).

Overall, these findings suggest that the Bicephali CAR architecture promotes more efficient immunological synapse formation, leading to enhanced proximal signaling activation, improved mitochondrial fitness, and ultimately more sustained anti‐tumor activity than that achieved with the conventional BB002 design.

### 2.4 Bicephali CAR‐T Cells Exhibit Superior Anti‐Tumor Activity Than Conventional BB002 CAR Construct in NSCLC Xenograft Mouse Models

**FIGURE 3:**
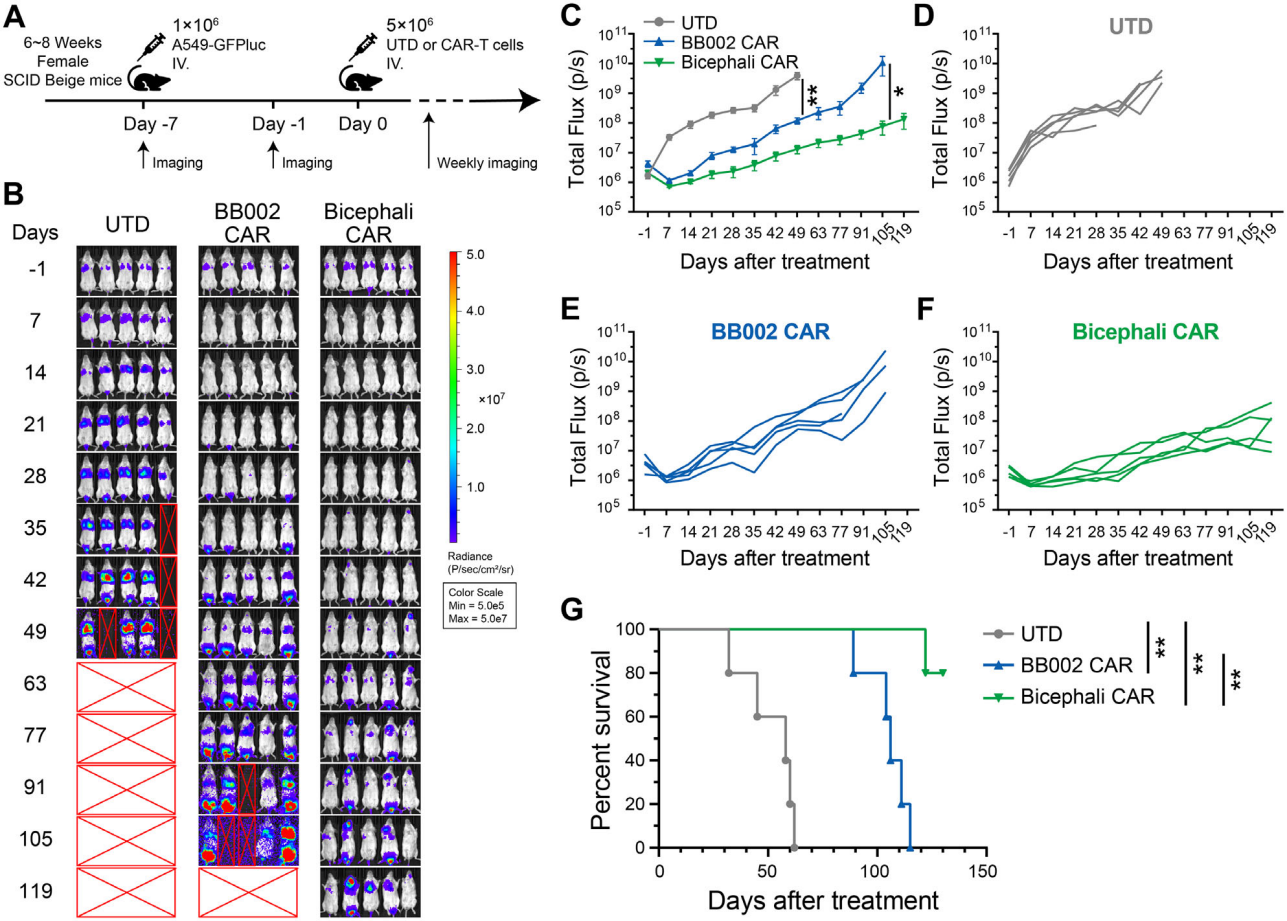
Bicephali CAR‐T cells exhibit superior anti‐tumor activity to conventional CAR construct in NSCLC xenograft mouse models. (A) Schematic diagram of the animal experiment design. (B) Representative bioluminescence images (BLI) of A549‐GFPluc tumor growth in the metastatic NSCLC model described in (A). (C) BLI kinetics of tumor growth as measured by total flux values (photons per second) at each time point. Data are presented as mean ± SEM of each group (n = 5) and the comparisons were analyzed using two‐way ANOVA with Tukey's multiple comparisons test. *p < 0.05 and **p < 0.01. (D–F) BLI kinetics of tumor growth for each individual mouse across experimental groups. n = 5 mice/group. (G) Kaplan–Meier survival curve of the mice in (B). The statistical significance of the difference was analyzed using the log‐rank (Mantel‐Cox) test. **p < 0.01.

To determine whether the greater in vitro cytotoxicity of Bicephali CAR‐T cells translated into enhanced anti‐tumor activity in vivo, we established a NSCLC xenograft model in SCID‐Beige mice via intravenously injecting GFPluc‐engineered A549 cells. Tumor burden was monitored longitudinally by bioluminescence imaging (Figure 3A). Mice receiving untransduced T cells showed rapid tumor progression, with mortality onset occurring within 40 days post tumor inoculation (Figure 3B–D). In contrast, all CAR‐T cell treatments significantly suppressed tumor growth (Figure 3B–F) and prolonged survival of mice (Figure 3G), although none completely eradicated the tumors or prevented metastatic progression. Bicephali CAR‐T cells nevertheless provided more durable tumor control, with less recurrence and longer‐term survival than conventional BB002 CAR‐T cells (Figure 3B–G). We further quantified circulating CAR‐T cells of tumor‐bearing mice after treatment on days 21 and 28 post infusion. Consistent with their enhanced memory phenotype under repeated antigen stimulation, Bicephali CAR‐T cells exhibited greater persistence in vivo, with higher peripheral‐blood frequencies and absolute counts at both time points (Figure S6).

Collectively, the in vitro and in vivo studies established that Bicephali CAR‐T cells provide more sustained anti‐tumor activity over conventional BB002 CAR‐T cells, with improved tumor control, survival, and in vivo persistence.

### 2.5 Bicephali CAR‐T Cells Show More Sustained Tumor Suppression Than Monospecific CAR‐T Cells in CD276/NKG2DLs Co‐Expressing NSCLC

Having demonstrated greater anti‐tumor activity of Bicephali CAR‐T cells compared to conventional BB002 CAR‐T cells, we next evaluated whether the Bicephali design could address antigen heterogeneity by comparison with monospecific CAR‐T cells.

**FIGURE 4:**
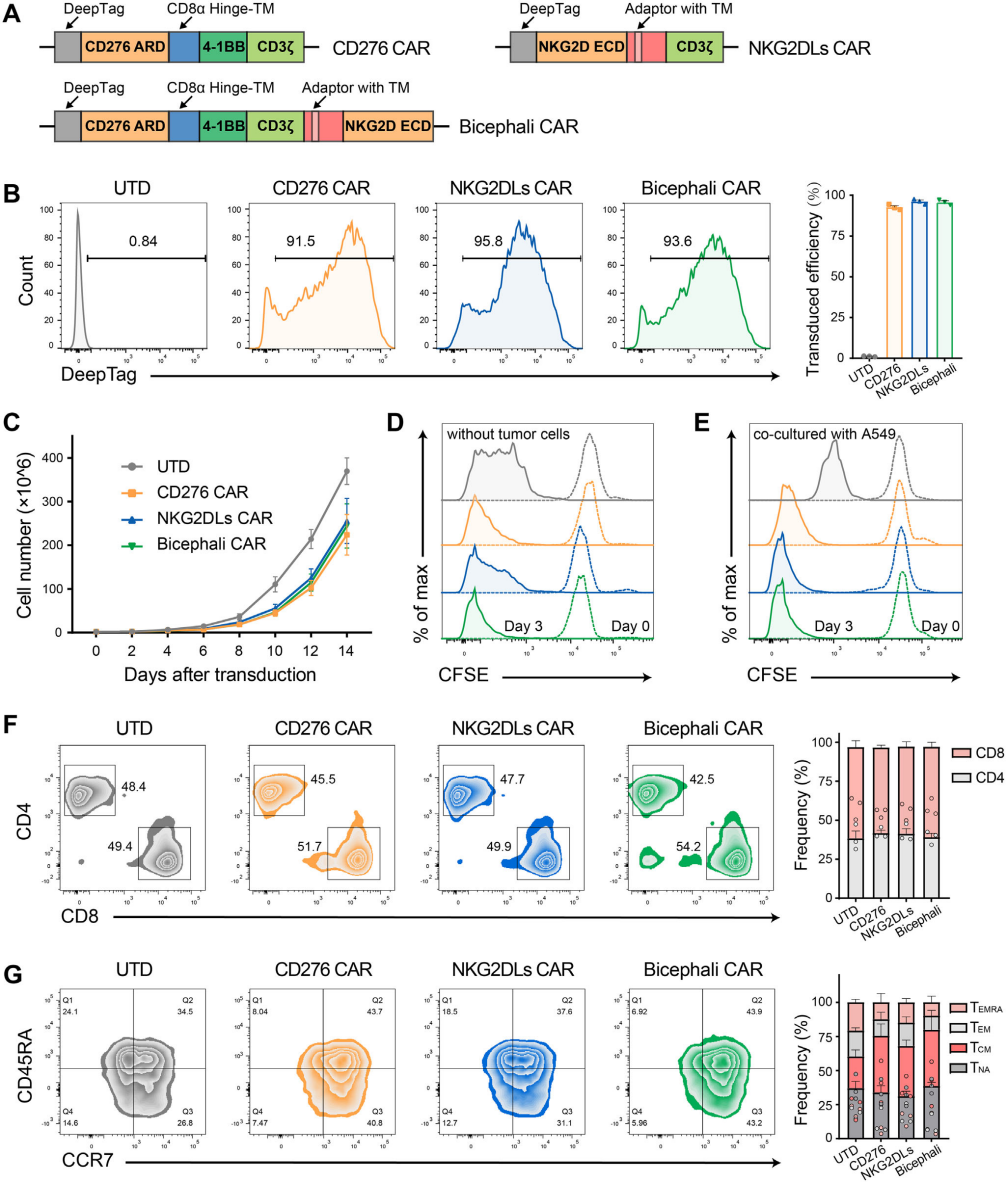
Generation and characterization of CD276/NKG2DLs monospecific and multi‐targeting Bicephali CAR‐T cells. (A) Schematic diagram of the CD276‐monospecific, NKG2DLs‐monospecific, and CD276/NKG2DLs multi‐targeting Bicephali CAR constructs. ARD, antigen recognition domain; ECD, extracellular domain; TM, transmembrane domain. (B) Transduction efficiency measured by flow cytometry and reported by the DeepTag. Data are presented as mean ± SEM of untransduced T (UTD) or CAR‐T cells generated from three independent donors. (C) Cell proliferation reflected by absolute cell counts of UTD and CAR‐T cells during 14 days of routine in vitro culture. The culture medium used was complete RPMI 1640 supplemented with 300 IU/mL human recombinant IL‐2. Data are presented as mean ± SEM of UTD or CAR‐T cells generated from three independent donors. (D) UTD and CAR‐T cells were labeled with carboxyfluorescein succinimidyl ester (CFSE), and their proliferation was assessed by flow cytometry after 3 days of culture without tumor‐cell stimulation. The culture medium used was complete RPMI 1640 supplemented with 300 IU/mL recombinant human IL‐2. Results from a representative donor (n = 3) are displayed. (E) CFSE‐labeled UTD and CAR‐T cells were co‐cultured with A549 tumor cells for 3 days, and proliferation was analyzed by flow cytometry to assess tumor antigen‐driven expansion. The culture medium used was complete DMEM suitable for A549 target cells without additional cytokines. Representative data from one of three donors are displayed. (F) Representative plots (left) and mean frequencies ± SEM (right) of CD4^+^/CD8^+^ ratios for UTD and CAR‐T cells generated from three independent donors. (G) Representative flow cytometry plots (left) and mean frequencies ± SEM (right) of naïve/stem cell‐like memory T cells (T_NA_, CD45RA^+^/CCR7^+^), central memory T cells (T_CM_, CD45RA^−^/CCR7^+^), effector memory T cells (T_EM_, CD45RA^−^/CCR7^−^), and terminally differentiated effector memory T cells (T_EMRA_, CD45RA^+^/CCR7^−^) in UTD and CAR‐T cells generated from three independent donors.

We generated monospecific CARs targeting CD276 or NKG2DLs individually and a multi‐specific Bicephali CAR targeting both antigen classes (Figure 4A). Lentiviral transduction of activated peripheral blood mononuclear cells (PBMCs) produced more than 90% surface CAR expression without impairing CAR‐T cell proliferative capacity (Figure 4B,C). CFSE dilution further showed that CAR‐T cells and untransduced T (UTD) cells proliferated comparably under routine culture conditions (Figure 4D). During co‐culture with A549 cells, however, CAR‐T cells showed a more pronounced leftward shift in CFSE fluorescence than UTD cells, indicating additional antigen‐driven proliferation (Figure 4E). CD4^+^/CD8^+^ ratios and memory‐subset composition were comparable across CAR‐T cell groups (Figure 4F,G).

**FIGURE 5:**
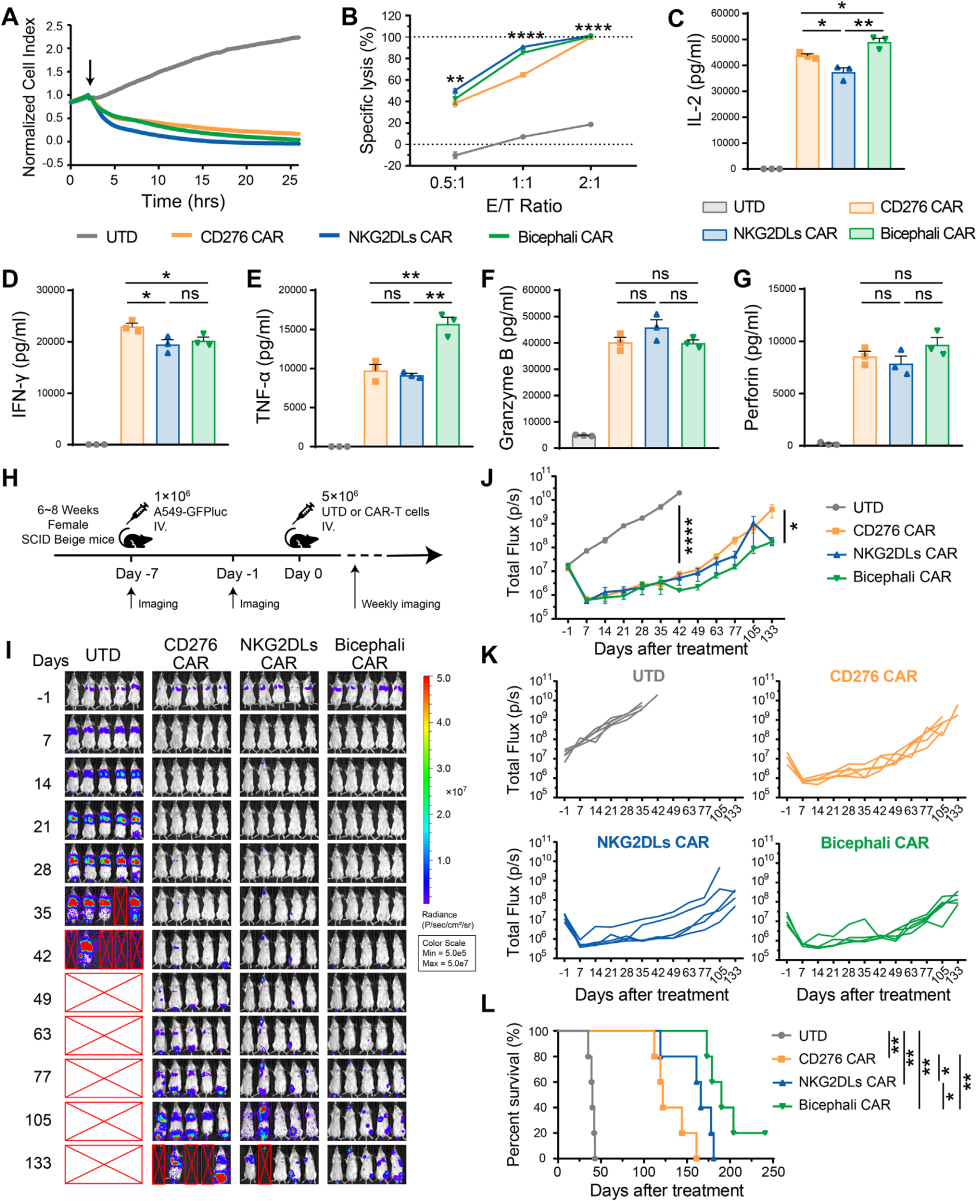
Bicephali CAR‐T cells outperform monospecific CAR‐T cells in long‐term tumor suppression against CD276/NKG2DLs co‐expressing NSCLC. (A) xCELLigence RTCA was used to monitor the cytolysis of human NSCLC tumor cell line A549 by CAR‐T cells. Effector‐to‐target (E:T) cell ratio = 1:1. Representative cytotoxicity data from three independent experiments using T cells from three healthy donors are shown. (B) Specific lytic ability of CAR‐T cells against the human NSCLC tumor cell line A549 at various E:T ratios obtained from the RTCA analysis. Data are presented as mean ± SEM of three replicates. Comparisons among groups were conducted by two‐way ANOVA with Tukey's multiple comparisons test. **p < 0.01 and ****p < 0.0001. (C–G) Analysis of the levels of cytokines IL‐2 (C), IFN‐γ (D), TNF‐α (E), granzyme B (F), and perforin (G) in the supernatants of untransduced T (UTD) or CAR‐T cells co‐cultured with A549 cells for 24 h. E:T ratio = 1:1. Data are presented as mean ± SEM of three replicates. Comparisons among groups were conducted using an unpaired, two‐tailed Student's *t*‐test. *p < 0.05 and **p < 0.01. ns, not significant. (H) Schematic diagram of the animal experiment design. (I) Representative bioluminescence images (BLI) of the A549‐GFPluc tumor growth in the metastatic NSCLC model described in (H). (J) BLI kinetics of tumor growth as measured by total flux values (photons per second) at each time point. Data are presented as mean ± SEM of each group (n = 5) and the comparisons were analyzed using two‐way ANOVA with Tukey's multiple comparisons test. *p < 0.05 and ****p < 0.0001. (K) BLI kinetics of tumor growth for each individual mouse. n = 5 mice/group. (L) Kaplan–Meier survival curve of the mice in (I). The statistical significance of the difference was analyzed using the log‐rank (Mantel‐Cox) test. *p < 0.05 and **p < 0.01.

Before evaluating efficacy under antigen heterogeneity, we first assessed the anti‐tumor activity of the CAR‐T constructs in tumor models with homogeneous antigen expression. RTCA assays showed that both monospecific and multi‐targeting Bicephali CAR‐T cells exhibited potent cytotoxicity against CD276 and NKG2DLs‐positive NSCLC cells (Figure 5A; Figure S7A,B), with dose‐dependent activity across the tested effector‐to‐target ratios (Figure 5B; Figure S7C,D). After 24 h of co‐culture with NSCLC cell lines, both monospecific and multi‐targeting Bicephali CAR‐T cells secreted significantly more IL‐2, IFN‐γ, TNF‐α, granzyme B, and perforin than untransduced T cells (Figure 5C–G; Figure S7E–I). Notably, Bicephali CAR‐T cells produced higher levels of IL‐2 and TNF‐α, consistent with greater effector activity.

In A549 NSCLC xenograft models (Figure 5H), CAR‐T treatment produced prolonged tumor suppression (Figure 5I–K) and markedly extended survival (Figure 5L) relative to untransduced T cells. Bicephali CAR‐T cells tended to provide more durable tumor control than monospecific CAR‐T cells, with delayed recurrence and improved long‐term survival (Figure 5I–L). No treatment‐related weight loss was observed (Figure S8A), and histopathological examination of the brain, heart, liver, spleen, and kidney showed no morphological abnormalities or inflammatory changes (Figure S8B), indicating no evidence of severe toxicity associated with anti‐CD276/NKG2DLs CAR‐T treatment in this model.

Collectively, both monospecific and Bicephali CAR‐T cells exhibited potent and dose‐dependent cytotoxicity against CD276^+^NKG2DLs^+^ NSCLC cells. Bicephali CAR‐T cells provided more sustained tumor control and longer‐term survival than monospecific CAR‐T cells, supporting further evaluation of this multi‐targeting approach in NSCLC.

### 2.6 Bicephali CAR‐T Cells Overcome Antigen Heterogeneity in NSCLC Cells

Antigen heterogeneity can permit antigen‐negative tumor cells, driving post‐treatment relapse and limiting CAR‐T efficacy in solid tumors. To evaluate Bicephali CAR‐T efficacy against heterogeneous CD276/NKG2DLs expression in NSCLC, we generated CD276‐knockout A549 cells using CRISPR‐Cas9 system (A549‐CD276 KO; Figure S9A). Since we were unable to identify or generate a human tumor cell line completely lacking all NKG2DLs, reflecting the multigene composition and complex, dynamic regulation of the NKG2D ligand family, we established a CD276^+^NKG2DLs^−^ model by expressing human CD276 in mouse Lewis lung carcinoma cells, which naturally lack human CD276 and NKG2DLs (LLC‐CD276^+^; Figure S9B,C).

**FIGURE 6:**
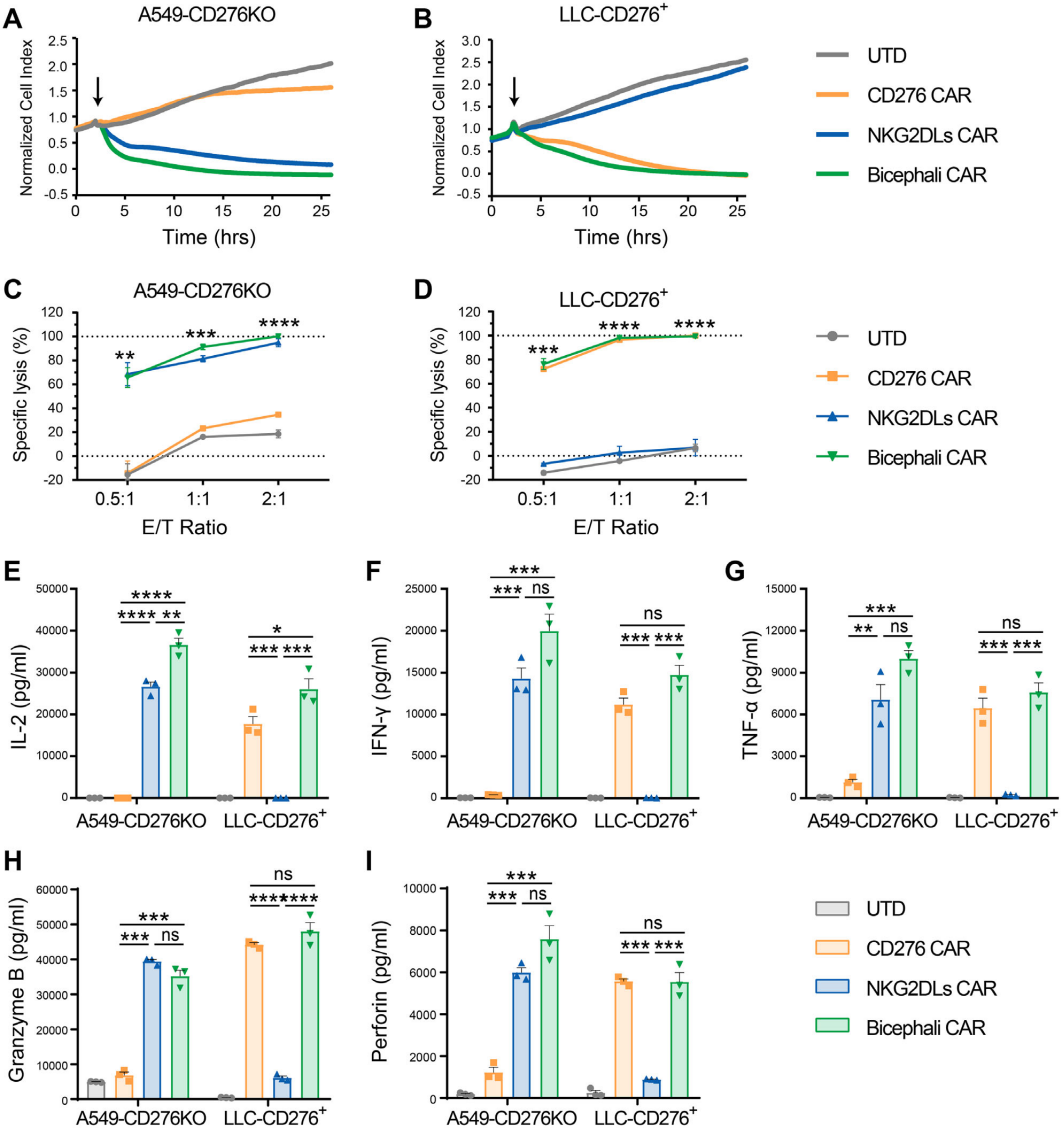
Bicephali CAR‐T cells overcome antigen heterogeneity in NSCLC cells. (A,B) xCELLigence RTCA was used to monitor the cytolysis of CD276^−^/NKG2DLs^+^ A549 (A) and CD276^+^/NKG2DLs^−^ LLC (B) tumor cells by CAR‐T cells. Effector‐to‐target (E:T) cell ratio = 1:1. Representative cytotoxicity data from three independent experiments using T cells from three healthy donors are shown. (C,D) Specific lytic ability of CAR‐T cells against CD276^−^/NKG2DLs^+^ A549 (C) and CD276^+^/NKG2DLs^−^ LLC tumor cells (D) at various E:T ratios obtained from the RTCA analysis. Data are presented as mean ± SEM of three replicates. Comparisons among groups were conducted by two‐way ANOVA with Tukey's multiple comparisons test. **p < 0.01, ***p < 0.001, and ****p < 0.0001. (E–I) Analysis of the levels of the cytokines IL‐2 (E), IFN‐γ (F), TNF‐α (G), granzyme B (H), and perforin (I) in the supernatants of untransduced T (UTD) or CAR‐T cells co‐cultured with CD276^−^/NKG2DLs^+^ A549 or CD276^+^/NKG2DLs^−^ LLC tumor cells for 24 h. E:T ratio = 1:1. Data are presented as mean ± SEM of three replicates. Comparisons among groups were conducted using an unpaired, two‐tailed Student's *t*‐test. P values are represented as either not significant (ns), *p < 0.05, **p < 0.01, ***p < 0.001, or ****p < 0.0001.

In vitro killing assays revealed that monospecific CAR‐T cells failed to lyse antigen‐negative NSCLC cells (Figure 6A–D). In contrast, Bicephali CAR‐T cells maintained potent, dose‐dependent cytotoxicity against these antigen‐loss NSCLC cells (Figure 6A–D). Moreover, Bicephali CAR‐T cells produced higher levels of T cell effector cytokines than monospecific CAR‐T cells when the corresponding target antigen was absent (Figure 6E–I). These findings show that simultaneous targeting of CD276 and NKG2DLs can maintain CAR‐T cell activity after loss of either antigen.

In addition, NKG2DLs‐targeting CAR‐T cells did not measurably impair the function of bystander NKG2D‐expressing immune cells, as NK‐cell degranulation, IFN‐γ production, and RTCA‐based tumor‐control activity were preserved in the presence of either active or irradiated CAR‐T cells, with irradiated CAR‐T cells serving as a non‐cytolytic competitor control (Figure S10). Similar findings were observed for human γδ T cells (Figure S11), supporting subsequent in vivo evaluation.

### 2.7 Bicephali CAR‐T Cells Overcome Antigen Heterogeneity in NSCLC Mouse Models

**FIGURE 7:**
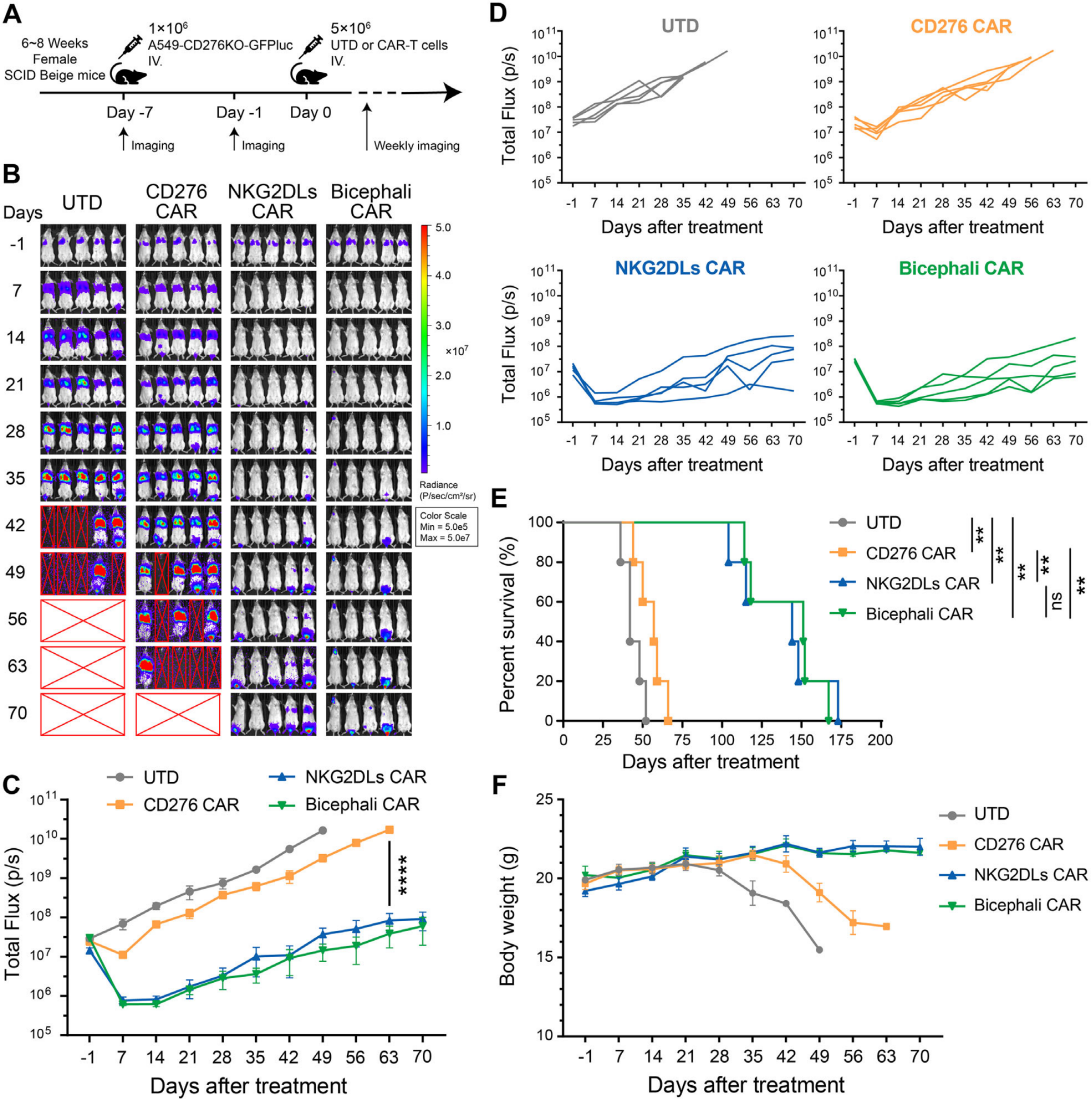
Bicephali CAR‐T cells overcome CD276 antigen loss in NSCLC xenograft mouse models. (A) Schematic diagram of the animal experiment design. (B) Representative bioluminescence images (BLI) of the A549‐CD276 KO‐GFPluc tumor growth in the metastatic NSCLC model described in (A). (C) BLI kinetics of tumor growth as measured by total flux values (photons per second) at each time point. Data are presented as mean ± SEM of each group (n = 5), and the results were compared using two‐way ANOVA with Tukey's multiple comparisons test. ****p < 0.0001. (D) BLI kinetics of tumor growth for each individual mouse. n = 5 mice/group. (E) Kaplan–Meier survival curve of the mice in (B). The statistical significance of the difference was analyzed using the log‐rank (Mantel‐Cox) test. ns, not significant. **p < 0.01. (F) Body weights of the mice in each group. Data are presented as mean ± SEM (n = 5).

**FIGURE 8:**
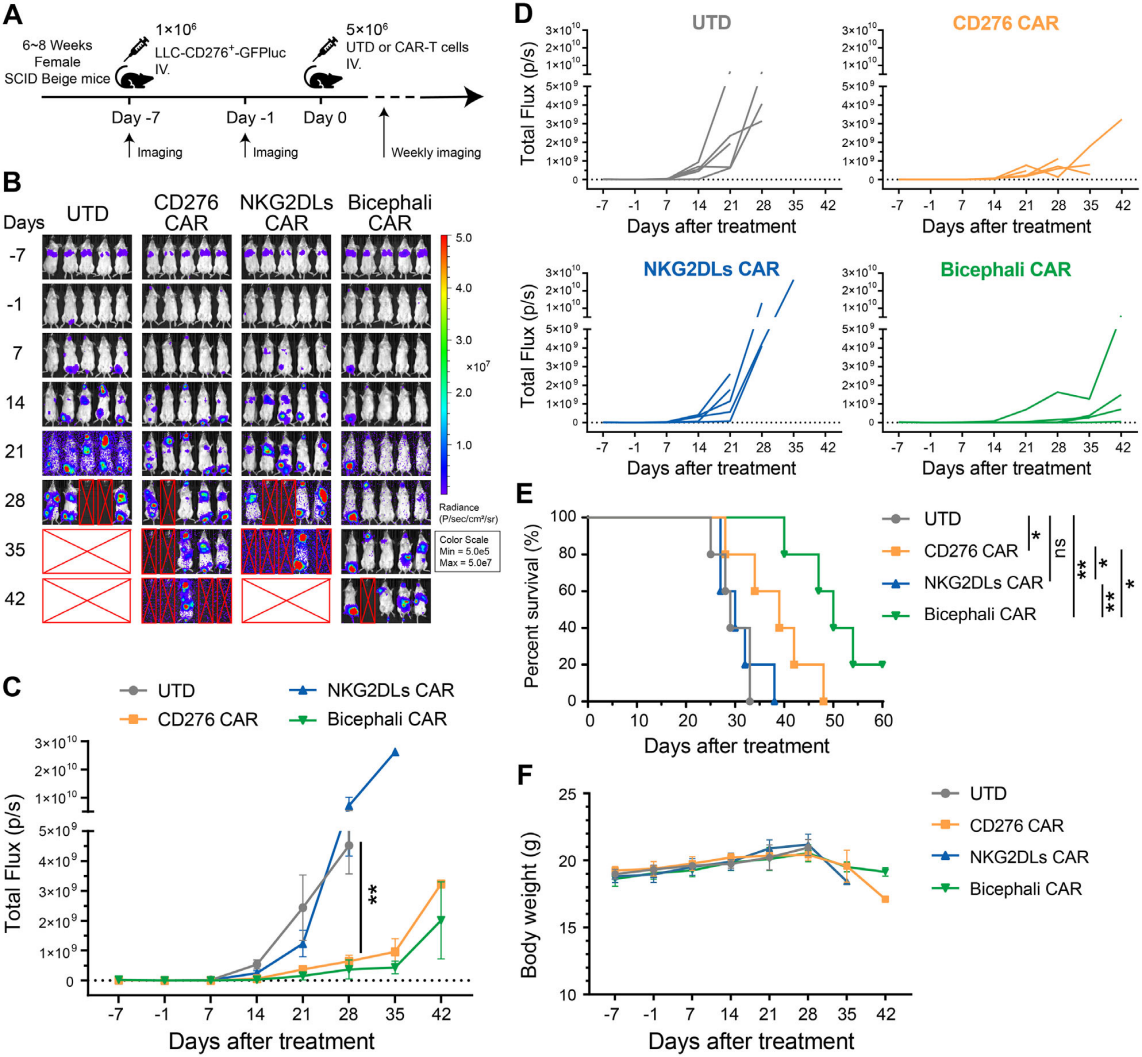
Bicephali CAR‐T cells overcome NKG2DLs antigen loss in NSCLC mouse models. (A) Schematic diagram of the animal experiment design. (B) Representative bioluminescence images (BLI) of the LLC‐CD276^+^‐GFPluc tumor growth in the metastatic NSCLC model described in (A). (C) BLI kinetics of tumor growth as measured by total flux values (photons per second) at each time point. Data are presented as mean ± SEM of each group (n = 5), and the results were compared using two‐way ANOVA with Tukey's multiple comparisons test. **p < 0.01. (D) BLI kinetics of tumor growth for each individual mouse. n = 5 mice/group. (E) Kaplan–Meier survival curve of the mice in (B). The statistical significance of the difference was analyzed using the log‐rank (Mantel‐Cox) test. ns, not significant. *p < 0.05 and **p < 0.01. (F) Body weights of the mice in each group. Data are presented as mean ± SEM (n = 5).

To determine whether Bicephali CAR‐T cells could prevent antigen escape in vivo, we established two complementary NSCLC mouse models in SCID‐Beige mice via intravenous injection of GFPluc‐engineered A549‐CD276 KO cells (Figure 7A) or GFPluc‐engineered‐LLC‐CD276^+^ cells (Figure 8A). Murine LLC cells showed no detectable human NKG2D ligands with the antibody panel used and, more importantly, were not functionally controlled by human NKG2DLs CAR‐T cells in vitro; they therefore provided an operational CD276‐positive/NKG2DLs‐unavailable model. Tumor‐bearing mice were subsequently treated with untransduced T cells, CD276 monospecific, NKG2DLs monospecific, or anti‐CD276/NKG2DLs Bicephali CAR‐T cells.

In the A549‐CD276 KO induced NSCLC xenograft mouse model, both Bicephali multi‐targeting CAR‐T and NKG2DLs‐monospecific CAR‐T cells cleared tumors in vivo (Figure 7B–D), whereas CD276‐monospecific CAR‐T cells failed to eliminate the tumor due to a lack of the target antigen (Figure 7B–D). Consistent with these findings, mice receiving Bicephali multi‐targeting CAR‐T or NKG2DLs‐monospecific CAR‐T cells exhibited a significantly prolonged survival compared with mice receiving UTD or anti‐CD276 CAR‐T therapy (Figure 7E). No treatment‐related weight loss was observed in any of the groups (Figure 7F).

In the LLC‐CD276^+^ NSCLC model, Bicephali CAR‐T cells delayed progression of NKG2DLs‐negative tumors significantly longer than NKG2DLs‐monospecific CAR‐T cells (Figure 8B–D). Neither CD276‐monospecific nor Bicephali CAR‐T cells completely eradicated tumors, potentially because of early systemic metastasis of LLC cells and the modest CAR‐T cell dose. Nevertheless, Bicephali CAR‐T cells achieved greater tumor control (Figure 8B–D) and prolonged survival (Figure 8E) relative to CD276‐monospecific CAR‐T cells. In addition, no treatment‐related weight loss was observed in any of the CAR‐T cell groups (Figure 8F).

Together, these findings show that Bicephali CAR‐T cells consistently overcome immune escape in heterogeneous tumor models, demonstrating critical advantages over single‐targeting approaches.

### 2.8 Bicephali CAR‐T Cells Sustain Anti‐Tumor Activity Through Enhanced Antioxidant Capacity, Reduced Exhaustion, and Preserved Stemness

To investigate the mechanisms underlying the greater durability of Bicephali relative to monospecific CAR‐T cells, we first performed bulk RNA sequencing after 24 h of co‐culture with A549 cells. Relative to untransduced T cells, CAR‐T cells presented broad transcriptomic changes (Figure S12A–C). Pathway enrichment analysis of differentially expressed genes (DEGs) identified increased cytokine‐receptor cross‐talk together with enrichment of TNF, JAK‐STAT, NF‐κB, and IL‐17 signaling pathways in CAR‐T cells compared with those in UTD (Figure S12D–F). These pathways are closely associated with T cell activation, proliferation, differentiation, and effector function.

**FIGURE 9:**
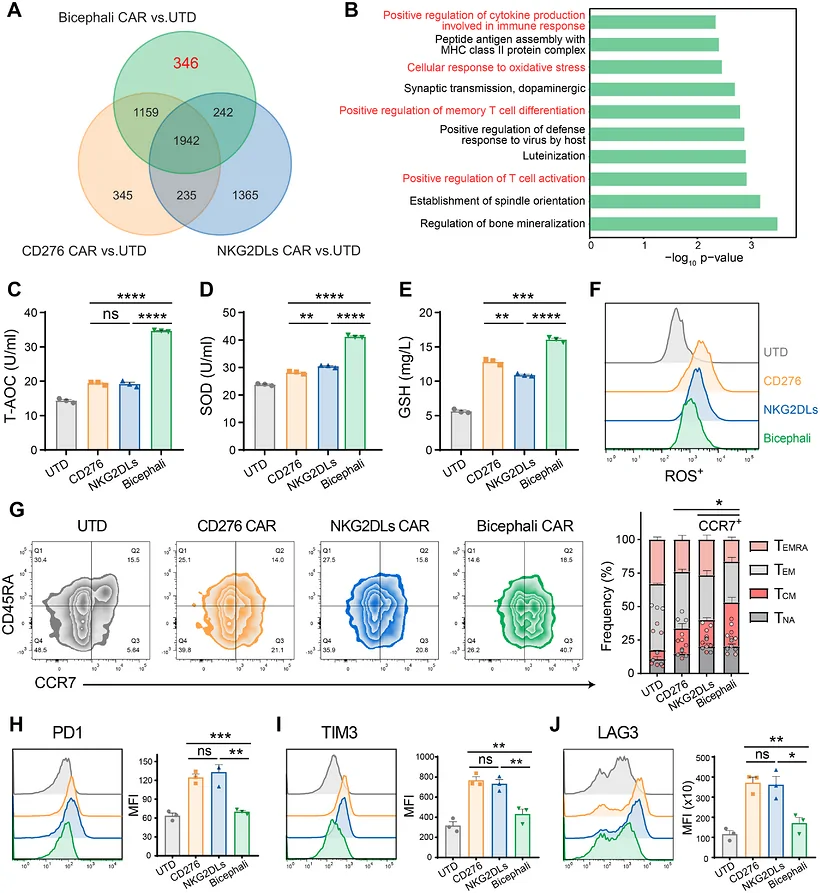
Bicephali CAR‐T cells alleviate oxidative stress, resist exhaustion, and preserve stemness. (A) Venn diagram showing unique and shared differentially expressed genes (DEGs) between CD276‐ or NKG2DLs‐targeting CAR‐T and multi‐targeting Bicephali CAR‐T cells stimulated by continuous antigen exposure (CAE). (B) Gene Ontology (GO) enrichment analysis of 346 unique DEGs in Bicephali CAR‐T cells depicted in (A). (C–E) Total antioxidant capacity (T‐AOC) (C), superoxide dismutase (SOD) (D) and glutathione (GSH) (E) content of CAR‐T cells after CAE. Data are presented as mean ± SEM of untransduced T (UTD) or CAR‐T cells generated from three independent donors. Comparisons among groups were conducted using an unpaired, two‐tailed Student's *t*‐test. **p < 0.01, ***p < 0.001, and ****p < 0.0001. ns, not significant. (F) Untransduced T (UTD) and CAR‐T cells were stained with DCFH‐DA and analyzed by flow cytometry. Results from a representative donor (n = 3) are displayed. (G) Representative flow cytometry plots (left) and mean frequencies ± SEM (right) for the proportions of naïve/stem cell‐like memory T cells (T_NA_, CD45RA^+^/CCR7^+^), central memory T cells (T_CM_, CD45RA^−^/CCR7^+^), effector memory T cells (T_EM_, CD45RA^−^/CCR7^−^), and terminally differentiated effector memory T cells (T_EMRA_, CD45RA^+^/CCR7^−^) in UTD and CAR‐T cells generated from three independent donors after 5 cycles of CAE. Differences between groups were assessed using an unpaired, two‐tailed Student's *t*‐test. *p < 0.05. (H–J) Exhaustion markers PD1 (H), TIM3 (I), and LAG3 (J) in CAR‐T cells following A549 CAE. Data are presented as mean ± SEM of untransduced T (UTD) or CAR‐T cells generated from three independent donors. Comparisons among groups were conducted using an unpaired, two‐tailed Student's *t*‐test. *p < 0.05, **p < 0.01, and ***p < 0.001. ns, not significant.

In addition to antigen heterogeneity, CAR‐T therapy in solid tumors is constrained by metabolic stress, including hypoxia and nutrient deprivation, together with immunosuppressive signals in the tumor microenvironment [35]. These conditions promote CAR‐T cell exhaustion and functional decline, reduce persistence, and limit the durability of anti‐tumor responses. We therefore used a CAE model to determine whether Bicephali CAR‐T cells resist exhaustion more effectively than monospecific CAR‐T cells. CAE produced extensive transcriptomic differences among the CAR‐T and UTD groups, with thousands of DEGs identified (Figure 9A). To define features specific to Bicephali CAR‐T cells, we compared the DEGs across the three CAR‐T groups and identified 346 genes unique to the multi‐targeting Bicephali CAR‐T cells (Figure 9A). Gene Ontology enrichment analysis of these genes highlighted pathways related to positive regulation of T cell function and oxidative stress (Figure 9B).

T cell activation and function depend on redox homeostasis. We further evaluated the redox status and functional capacity of CAR‐T cells. Bicephali CAR‐T cells exhibited increased total antioxidant capacity, elevated superoxide dismutase and glutathione levels (Figure 9C–E), and reduced intracellular reactive oxygen species (ROS) accumulation (Figure 9F), indicating enhanced antioxidant capacity. Prior studies have demonstrated that antioxidant capacity ameliorates CAR‐T cell exhaustion and drives long‐lived memory CAR‐T cells [36, 37]. We then assessed memory differentiation of CAR‐T cells after CAE. Relative to monospecific CAR‐T cells, Bicephali CAR‐T cells retained a less differentiated phenotype, with higher proportions of naïve and central memory subsets (CCR7^+^) (Figure 9G). Expression of exhaustion markers (PD1, TIM3, and LAG3) across the CAR‐T cell groups was lower in Bicephali CAR‐T cells (Figure 9H–J), consistent with reduced exhaustion.

In summary, Bicephali CAR‐T cells achieved sustained anti‐tumor activity in immunosuppressive microenvironments through enhanced antioxidant defenses that maintained redox balance, mitigated exhaustion, and preserved stem‐like differentiation states.

## 3 Discussion

CAR‐T cell therapy for solid tumors remains limited by several factors, including antigen heterogeneity, CAR‐T cell exhaustion, and poor persistence [7, 38]. Strategies under investigation include combinatorial antigen targeting, additional immunomodulatory components, and modulation of the tumor microenvironment [38, 39, 40, 41]. In this study, CD276 and NKG2DLs were identified as heterogeneously expressed targets in NSCLC, and a novel Bicephali CAR‐T platform was developed to recognize both antigen classes. The resulting multi‐targeting CAR‐T cells showed improved tumor control in models of antigen heterogeneity and maintained functional activity during repeated antigen exposure.

The optimal target antigens for CAR‐T therapy in NSCLC remain under active investigation, among which CD276 holds great clinical potential. CD276 is expressed in a high proportion of NSCLC cases, shows a relatively tumor‐restricted expression pattern, and has been associated with poor clinical prognosis [25, 42]. Preclinical studies have reported anti‐tumor activity of CD276‐specific CAR‐T cells in NSCLC models [43, 44]. Consistent with these reports, CD276‐monospecific CAR‐T cells exhibited potent and dose‐dependent cytotoxicity against NSCLC cells and substantially prolonged survival in tumor‐bearing mice [43, 44]. However, this activity was abolished when the CD276 antigen was absent, illustrating the limitation of monospecific targeting in tumors with heterogeneous antigen expression in NSCLC and supporting the development of multi‐targeting strategies.

NKG2DLs represent another clinically relevant target class in NSCLC and show preferential expression in malignant tissues. Pharmacological upregulation of NKG2DLs has been reported to enhance anti‐tumor responses in osteosarcoma and lung cancer models [45, 46]. NKG2DLs‐targeting CAR‐T cells have shown activity in triple‐negative breast cancer [47], hepatocellular carcinoma [16], pancreatic carcinoma [48], and glioblastoma [17]. In this study, the extracellular domain of native NKG2D was used as the targeting moiety, preserving recognition of multiple NKG2D ligands. NKG2DLs‐targeting CAR‐T cells showed greater in vivo tumor control and survival than CD276‐ targeting CAR‐T cells in the models examined. Together with the antigen heterogeneity findings, these results support concurrent targeting of CD276 and NKG2DLs as a strategy to reduce antigen escape in NSCLC.

Dual‐targeting and multi‐specific CAR‐T strategies are being actively explored to reduce relapses associated with antigen escape in heterogeneous tumors [49]. Beyond target selection, the optimal structural configuration for integrating two recognition modules within a single CAR remains incompletely defined. In this study, we present “Bicephali” as a novel multi‐targeting CAR design characterized by two completely distinct antigen‐binding and transmembrane domains that converge into a shared intracellular 4‐1BB/CD3ζ signaling cassette. This design enables streamlined activation against multiple antigens and mitigates the risk of steric interference in conventional dual‐scFv CAR constructs. Importantly, the final Bicephali construct was selected through receptor‐positioning optimization rather than arbitrary assembly. Placement of the NKG2D ECD at C terminus improved CAR expression and cytotoxicity, consistent with the native type II topology of NKG2D, in which the ligand‐binding domain is naturally oriented at C terminus. These findings underscore the importance of receptor topology in determining the performance of multi‐targeting CAR‐T cells. By integrating positional optimization with expanded antigen recognition, the Bicephali platform provides a refined architectural framework for next‐generation CAR design.

The structural interpretation is further supported by subsequent mechanistic analysis, which suggest that the functional advantage of Bicephali design arises upstream of the shared 4‐1BB/CD3ζ signaling module, at the level of target cell engagement. Compared with conventional BB002 construct, Bicephali CAR‐T cells formed more organized immunological synapses and showed greater ZAP70 and PLCγ phosphorylation upon tumor cell stimulation, consistent with enhanced proximal signaling. These findings were accompanied by improved mitochondrial fitness, reduced ROS accumulation, and enhanced antioxidant capacity. Mitochondrial function is increasingly recognized as an important determinant of CAR‐T cell memory differentiation, expansion, persistence, and long‐term therapeutic efficacy [50, 51]. By contrast, metabolic restrictions (e.g. hypoxia and nutrient deprivation) in the solid tumor microenvironment can compromise CAR‐T cell metabolism and promote exhaustion [33]. Approaches that restore mitochondrial function or reduce ROS accumulation can improve CAR‐T cell function [37, 52, 53]. Transcriptomic analysis further showed activation of cytokine‐receptor cross‐talk and NF‐κB, JAK‐STAT, and IL‐17 signaling pathways, which are involved in inflammatory and immune responses [54, 55]. These pathways may contribute to CAR‐T cell effector function and influence other immune cells within the tumor microenvironment [39, 56]. Bicephali CAR‐T cells secreted higher levels of IL‐2 and TNF‐α. IL‐2 supports T cell proliferation, effector differentiation, and memory cell generation [57], whereas TNF‐α contributes to inflammatory responses and T cell persistence [58]. Together, these findings support a coordinated mechanistic framework in which the Bicephali architecture enhances synapse assembly, strengthens proximal signaling, preserves mitochondrial and redox homeostasis, and sustains effector cytokine production, thereby promoting more durable CAR‐T cell activity.

Limited expansion and persistence after infusion remain important barriers to CAR‐T cell efficacy in solid tumors [36]. Current CAR‐T architectures that employ excessive co‐stimulatory and signaling modules exhibit an inherent vulnerability to inducing signal interference and T‐cell hyperactivation, consequently increasing the risk of T cell exhaustion [41, 59]. In the continuous antigen exposure model, Bicephali CAR‐T cells maintained anti‐tumor activity while preserving less differentiated memory phenotypes and showing lower expression of exhaustion markers. Less differentiated subsets, particularly naïve/stem cell‐like memory T cells (T_NA_) and central memory T cells (T_CM_), are associated with greater proliferative capacity, prolonged survival, and durable anti‐tumor responses [60]. Consistent with these findings, mice treated with Bicephali CAR‐T cells demonstrated significantly delayed tumor progression and postponed recurrence compared to those treated with monospecific CAR‐T cells in this study.

In conclusion, we developed a novel therapeutic strategy using the Bicephali CAR‐T platform to simultaneously target CD276 and NKG2DLs and reduce susceptibility to antigen loss in preclinical NSCLC models. Bicephali CAR‐T cells showed improved immunological synapse organization, mitochondrial fitness, antioxidant capacity, and persistence, together with sustained anti‐tumor activity in homogeneous and antigen‐heterogeneous models. These preclinical findings support further evaluation of this multi‐targeting strategy in NSCLC and other solid tumors with heterogeneous antigen expression.

## 4 Methods

### 4.1 Cell Lines and Culture Condition

Human LUAD cell lines A549 (CCL‐185, RRID: CVCL_0023) and NCI‐H1299 (CRL‐5803, RRID: CVCL_0060), human large cell lung cancer cell line NCI‐H460 (HTB‐177, RRID: CVCL_0459), human LUSC cell line SK‐MES‐1 (HTB‐58, RRID: CVCL_0630), mouse Lewis lung carcinoma cells (LLC; CRL‐1642, RRID: CVCL_4358), and human embryonic kidney 293T cells (CRL‐3216, RRID: CVCL_0063) were obtained from the American Type Culture Collection (ATCC) and cultured according to the provided instructions. Human lung adenocarcinoma cell line PC9 was obtained from Procell (CL‐0668, RRID: CVCL_B260). NCI‐H1299, PC9, and NCI‐H460 cells were cultured in RPMI‐1640 medium (Gibco), whereas A549, SK‐MES‐1, LLC, and 293T cells were cultured in DMEM medium (Gibco). Media were supplemented with 10% fetal bovine serum (FBS) (Gemini Bio‐Products), 1% penicillin/streptomycin (Gibco), and 1% GlutaMAX (Gibco). Cells were maintained at 37°C in a humidified incubator with 5% CO_2_. All cell lines were authenticated by short tandem repeat (STR) profiling, with no evidence of cross‐contamination or misidentification. Cell passage numbers were monitored and maintained within 10 passages after thawing throughout the study, and cultures were routinely screened to confirm the absence of mycoplasma contamination.

### 4.2 Generation of Cell Lines

To enable cell tracking, A549 and LLC cells were transduced with a lentiviral vector encoding the GFP‐firefly luciferase (GFPluc) reporter. CD276 was disrupted in A549 cells by electroporation‐mediated delivery of CRISPR‐Cas9 and a target‐specific sgRNA (CAGCTACCGGGGCTACCCTG) using the Celetrix electroporation system (Celetrix, Manassas, VA, USA). Stable LLC cells overexpressing human CD276 were generated by lentiviral transduction.

### 4.3 Immunohistochemistry

Tissue microarrays of human NSCLC and multiple normal organs were purchased from Xi'an bioaitech Co., Ltd. (Xi'an, China). IHC was performed as described previously [61]. Briefly, the tissue microarray sections were deparaffinized in xylene and rehydrated through a graded ethanol series. Antigen retrieval was performed in citrate buffer (pH 6.0) using two 5‐min microwave‐heating cycles. Endogenous peroxidase activity was blocked with 3% hydrogen peroxide in methanol for 10 min at room temperature. Slides were incubated overnight at 4°C with primary antibodies against human B7‐H3 (dilution 1:5000, Cat# 66481‐1‐Ig, Proteintech), ULBP‐2/5/6 (10 µg/mL, Cat# AF1298, R&D Systems), and MICA/MICB (15 µg/mL, Cat# MAB13001, R&D Systems), followed by a 10‐min of incubation with horseradish peroxidase (HRP)‐conjugated secondary antibodies. DAB staining was controlled under a microscope, and sections were counterstained with hematoxylin. Images were acquired using a ScanScope CS scanner (Aperio, USA). Two experienced pathologists independently evaluated the staining and reached a final consensus. Staining intensity was graded semiquantitatively as 0 (negative), 1 (weak), 2 (moderate), or 3 (strong positive), respectively [62, 63].

### 4.4 Lentivirus Production

A CD276 and NKG2DLs multi‐targeting CAR vector was constructed using the Bicephali platform developed by Shanghai YaKe Biotechnology Ltd. (patent pending). The lentiviral vector, named DeepTag‐CD276‐NKG2DL Bicephali, encodes a CAR comprising (1) a truncated extracellular BCMA (B‐cell maturation antigen), termed DeepTag (patent pending), fused to a CD276‐binding domain and served as a flow cytometry detection tag; (2) a CD8α hinge‐transmembrane domain; (3) 4‐1BB and CD3ζ signaling domains; and (4) an NKG2D extracellular domain linked through a proprietary adapter peptide to a secondary transmembrane domain. This architecture enables simultaneous targeting of CD276 and eight NKG2D ligands through a single chimeric antigen receptor.

For lentiviral packaging, pMD2.G envelope, gag‐pol packaging, and CAR‐encoding plasmids were co‐transfected into HEK293T cells using the chemical DNA transfection reagent PEI (Cat# 40‐872‐7, Sigma). Lentiviral supernatants were harvested at 48 and 72 h after transfection, followed by filtration and concentration via high‐speed ultracentrifugation. The resulting lentivirus was aliquoted and stored at −80°C.

### 4.5 Transduction and Expansion of CAR‐T Cells

Human PBMCs were isolated from healthy donor‐derived buffy coats commercially procured from YAYU Bio, which obtained written informed consent from all donors under a collection protocol approved by the Institutional Review Board of Shanghai Liquan Hospital (Approval No. SHLQ‐YLK‐2022‐09). Before transduction, PBMCs were activated with CD3/CD28 beads (Cat# 40203D, Thermo Fisher Scientific) at a bead‐to‐T‐cell ratio of 2:1. Cells were cultured in complete RPMI 1640 medium supplemented with 300 IU/mL recombinant human IL‐2. After 24 h of activation, PBMCs were transduced with CAR‐encoding lentiviral vectors at a multiplicity of infection of 15. Transduction Reagent (YaKe) was added at a ratio of 1:200 to facilitate lentiviral delivery. The following day, the medium was replaced with fresh complete RPMI 1640 medium containing 300 IU/mL IL‐2. Transduced T cells were expanded, maintaining a cell density of 0.5–1.0 × 10^6^ cells/mL. Transduction efficiency was assessed by flow cytometry on day 6 post‐transduction and reconfirmed before downstream analysis. Dynabeads were removed by magnetic separation after 7 days. To normalize the number of CAR‐positive T cells across constructs for functional assays, UTD T cells were added to dilute the percentage of CAR^+^ cells to the lowest transduction efficiency. All functional assays were performed within 7–14 days post‐transduction.

### 4.6 RTCA Based Cytotoxicity Assays

CAR‐T cell cytotoxicity was assessed using the xCELLigence RTCA system (Agilent, California, USA). Human NSCLC cells were seeded at 1 × 10^4^ cells/well in 96‐well E‐Plates. After 24 h, CAR‐T cells were added at the indicated effector‐to‐target (E:T) ratios in the standard medium for each target cell line without exogenous cytokines. Cells were maintained at 37°C with 5% CO_2_ for at least 24 h, and impedance‐based normalized cell index (NCI) measurements were recorded every 15 min. Data were acquired and analyzed using xCELLigence RTCA Software Pro 2.6.1. The NCI plot internally divides the cell index for every selected well by the cell index at the normalization time point, which corresponds to the time when effectors are added to samples. Specific lysis at 24 h after co‐culture was calculated as follows: lysis ratio (%) = (1‐(NCI_treatment_/NCI_target only_)) × 100% [64].

### 4.7 Analysis of Cytokine Production

For cytokine measurements, 2 × 10^4^ CAR‐T cells were co‐cultured with target cells (E:T = 1:1) in 200 µL of complete medium without exogenous cytokines in a 96‐well plate. Supernatants were collected after 24 h at 37°C, and cytokine concentrations were measured using bead‐based LEGENDplex immunoassays (Cat# 741187, BioLegend) according to the manufacturer's protocol.

### 4.8 Flow Cytometry

Cells were harvested, washed twice with 1× PBS, and resuspended in cold PBS containing 2% FBS at a concentration not exceeding 1 × 10^6^ cells/mL. Subsequently, the flow cytometry antibodies were added to the cell suspension, and the mixture was incubated for 20 min at 4°C in the dark according to the manufacturer's instructions. CD276 and NKG2D ligands expression on NSCLC cell lines was assessed using APC‐conjugated mouse anti‐human CD276 (B7‐H3) (Cat# 351006, BioLegend), PE‐conjugated mouse anti‐human ULBP1 (Cat# FAB1380P, R&D Systems), ULBP‐2/5/6 (Cat# FAB1298P, R&D Systems), ULBP3 (Cat# FAB1517P, R&D Systems), ULBP4 (Cat# FAB6285P, R&D Systems), and MICA/MICB (Cat# FAB13001P, R&D Systems). CAR expression was measured using PE‐conjugated or BV421‐conjugated mouse anti‐human CD269 (BCMA) antibodies (Cat# 357503 and 357519, BioLegend) or a BV510‐conjugated mouse anti‐human NKG2D antibody (Cat# 563266, BD). CAR‐T cell differentiation and exhaustion were assessed using APC anti‐CD3 (555335), PE anti‐CD4 (555347), FITC anti‐CD8 (555366), PE‐CF594 anti‐CD8 (562282), APC anti‐CD45RA (550855), FITC anti‐CCR7 (561271), and APC anti‐CD279 (PD‐1) (558694), all purchased from BD Biosciences; Alexa Fluor 700 anti‐CD3 (300424), APC/Cy7 anti‐CD3 (344818), APC/Cy7 anti‐CD4 (300518), and PE anti‐CD223(LAG‐3) (369305), all purchased from BioLegend; and Alexa Fluor 647 anti‐TIM‐3 (FAB2365R), from R&D Systems. Zombie Aqua Live/Dead Staining (Cat# 423101, BioLegend) was utilized to identify viable cells. Isotype‐matched control mAbs were included in all procedures. At least 10 000 events per sample were acquired on a BD FACSCelesta (BD Biosciences) and analyzed using FlowJo v10.8.1.

### 4.9 CFSE Proliferation Assay

1 day before co‐culture, A549 cells were seeded in six‐well plates at 5 × 10^5^ cells/well. CAR‐T cells were labeled with 1.0 mM carboxyfluorescein diacetate succinimidyl ester (CFSE; Invitrogen) and co‐cultured with tumor cells at an E:T ratio of 1:1 for 72 h. CFSE dilution was then quantified by flow cytometry in gated CD3^+^ or CAR^+^ T cell populations.

### 4.10 Confocal Imaging of Immunological Synapse

CellTracker Green CMFDA‐labeled CAR‐T cells were mixed with A549 cells in 200 µL of T cell medium and incubated for 10 min at 37°C. The cell mixture was then gently transferred to silane‐coated glass slides and incubated for an additional 10 min at 37°C [65]. Cells were fixed with 4% formaldehyde, permeabilized with Triton X‐100, and incubated with primary antibodies overnight at 4°C. To visualize the IS, F‐actin was stained with phalloidin‐iFluor 594 (Cat# 40786ES75, YEASEN), and the microtubule organizing center (MTOC) was detected using an anti‐pericentrin antibody (Cat# S0B6376, STARTER) followed by an Alexa Fluor 647‐conjugated secondary antibody. Nuclei were stained with DAPI. Confocal images were acquired using a Zeiss LSM 800 confocal laser scanning microscope. Images were processed and analyzed with ZEN Lite 3.8 (Zeiss).

### 4.11 Western Blot

CAR‐T cells were stimulated with A549 cells at an E:T ratio of 2:1 for the indicated durations. Reactions were terminated by adding 2× lysis buffer containing a protease inhibitor cocktail (Cat# HY‐K0010, MCE) and a phosphatase inhibitor cocktail (Cat# HY‐K0022, MCE), followed by incubation on ice for 30 min. Lysates were centrifuged, and the supernatants were collected. For immunoblotting, 20 µg of protein was separated by SDS‐PAGE gels and transferred to PVDF membranes. Membranes were blocked and sequentially incubated with primary and secondary antibodies. Chemiluminescent signals were developed with ECL substrate (Cat# K1233, APExBIO) and captured using a ChemiDoc Imaging System (Bio‐Rad) with exposure times of 1–60 s to ensure signal linearity.

The following primary antibodies were used: phospho‐PLCγ1 (Tyr783) (Cat# 14008, CST), PLCγ1 (D9H10) (Cat# 5690, CST), phospho‐Zap‐70 (Tyr319)/Syk (Tyr352) (Cat# 2717, CST), Zap‐70 (D1C10E) (Cat# 3165, CST), phospho‐ATF‐2 (Thr71)/ATF7 (Thr53) (Cat# 27934, CST), ATF‐2 (D4L2X) (Cat# 35031, CST), Mitofusin‐2 (Cat# 9482, CST), and beta‐Actin (Cat# 4967, CST).

### 4.12 Continuous Antigen Exposure

CAR‐T cell exhaustion was induced using a CAE model described previously, with minor modifications [66]. Briefly, A549 cells were seeded into six‐well plates at 1 × 10^6^ cells/well 1 day before addition of CAR‐T cells. CAR‐T cells were added at an E:T ratio of 1:2. After 2–3 days, co‐culture supernatants containing CAR‐T cells were collected and centrifuged. Recovered CAR‐T cells were resuspended, counted, and replated with fresh A549 cells (E:T = 1:2) for subsequent rounds. Following five cycles, CAR‐T cells were harvested for analysis.

### 4.13 Antioxidant Enzyme Assays

CAR‐T cells stimulated with A549 cells were collected, washed with PBS, and sonicated on ice to generate cell lysates. Total antioxidant capacity (T‐AOC), superoxide dismutase (SOD) activity, and glutathione levels were measured using commercial assay kits (Nanjing Jiancheng Bioengineering Institute, China) according to the manufacturer's protocols.

### 4.14 Reactive Oxygen Species Measurement

CAR‐T cells stimulated with A549 cells were collected, washed with PBS, and incubated with 10 µM DCFH‐DA (Sigma‐Aldrich, D6883) for 1 h at 37°C. The cell‐permeable, non‐fluorescent probe DCFH‐DA is hydrolyzed by intracellular esterases to DCFH and subsequently oxidized by ROS to fluorescent DCF. Intracellular ROS levels were quantified by flow cytometry based on 488nm‐excited fluorescence intensity.

### 4.15 Animal Experiments

6‐ to 8‐week‐old female SCID‐Beige mice were purchased from SLAC Laboratory Animal Co. Ltd. (Shanghai, China) and maintained under specific‐pathogen‐free conditions at the Experimental Animal Center of Shanghai Ninth People's Hospital. All animal experiments were conducted under protocols approved by the Institutional Animal Care and Use Committee of Shanghai Ninth People's Hospital (No. SH9H‐2024‐A1276‐1).

To establish human NSCLC metastatic models, mice were intravenously injected with 1 × 10^6^ GFPluc‐engineered A549 cells co‐expressing CD276 and NKG2D ligands. 7 days post‐inoculation, mice were randomly assigned to treatment groups using a computer‐generated randomization sequence, with baseline bioluminescence signals balanced across groups to ensure comparable tumor burdens, followed by intravenous administration of untransduced T cells or CAR‐T cells (5 × 10^6^ cells/mouse). For antigen‐heterogeneous models, CD276‐knockout A549 cells and human CD276‐overexpressing LLC cells, which intrinsically lack human CD276 and NKG2D ligands, were engrafted following the same procedure.

Orbital blood was collected to quantify CAR‐T cell persistence by flow cytometry using absolute counting beads (Cat# C36995, Invitrogen). Tumor growth was monitored by bioluminescence imaging (BLI) using the IVIS Lumina II in vivo imaging system with Living Image software v4.5.2 (PerkinElmer). Mice received intraperitoneal D‐luciferin (150 mg/kg) once weekly, and radiance was quantified after auto‐mode imaging as total photons/sec/cm^2^/sr (p/s/cm^2^/sr). Investigators performing bioluminescence imaging, tumor measurements, and survival monitoring were blinded to group allocation. Throughout the experiments, body weight and general health status were closely monitored, and animals were daily assessed for signs of distress or clinically overt disease. Animals were euthanized upon showing signs of clinically overt disease or severe distress, including reduced food intake, decreased activity, abnormal grooming behavior, or hunched posture, or when excessive weight loss (15% body‐weight loss over a week) was observed.

### 4.16 Seahorse Assay

CAR‐T cells stimulated with A549 tumor cells were collected and washed in XF RPMI Base Medium supplemented with glucose (10 mM), sodium pyruvate (1 mM), and glutamine (2 mM). Cells were seeded at 2 × 10^5^ cells/well in Cell‐Tak (Corning)–coated XFe96 Cell Culture Microplates (Agilent). Following adherence and equilibration, oxygen consumption rate (OCR; pmol/min) was measured using an XF Cell Mito Stress Test kit (Agilent) with sequential addition of oligomycin (1.5 µM), carbonyl cyanide 4‐(trifluoromethoxy) phenylhydrazone (FCCP; 2 µM), and antimycin A plus rotenone (0.5 µM). All reagents were purchased from Agilent, and measurements were obtained using an Agilent Seahorse XFe Analyzer. Spare respiratory capacity was calculated as maximal OCR minus basal OCR.

### 4.17 RNA Sequencing

CAR‐T cells were co‐cultured with A549 tumor cells for 24 h at an E:T ratio of 1:1. Cell suspensions were then harvested, and CAR‐T cells were isolated using biotin‐conjugated anti‐BCMA mAbs (Cat# 357514, BioLegend) and anti‐biotin magnetic beads (Cat# 480016, BioLegend). Total RNA was extracted using RNA Extraction Reagent (Cat# R401‐01, Vazyme) according to the manufacturer's instructions. RNA quality assessment and transcriptome sequencing were conducted by Oebiotech (Shanghai, China). Bioinformatic analysis was performed using the OECloud platform (https://cloud.oebiotech.com).

### 4.18 TCGA Data Availability and Analysis

mRNA expression data for CD276 and NKG2DLs in the TCGA LUAD and TCGA LUSC cohorts were obtained from the USCS Xena portal (https://xena.ucsc.edu/) as log2(norm_count+1) values. The analysis included 1016 NSCLC tumor tissues (514 LUAD and 502 LUSC) and 110 normal lung tissues. CD276‐high and CD276‐low groups were defined using the median CD276 expression value in the NSCLC cohort. For NKG2DLs, a ligand‐family‐based classification was used because the NKG2D extracellular domain can recognize any of the eight human NKG2D ligands. Each ligand was first classified as high or low relative to its respective median expression value. A tumor was classified as NKG2DLs‐high when at least one ligand exceeded its respective median and as NKG2DLs‐low only when all eight ligands were below their respective medians. The original TCGA expression matrix and subgroup‐classification table are provided as Supporting File 2.

### 4.19 NK Cell Expansion

PBMCs were depleted of CD3^+^ T cells using Dynabeads CD3 (Cat# 11151D, Invitrogen) at a bead‐to‐PBMC ratio of 3:1 according to the manufacturer's instructions. The remaining cells were co‐cultured with artificial antigen‐presenting cells (aAPCs) at a 1:1 ratio to initiate NK cell induction and expansion and maintained at 37°C in a humidified incubator with 5% CO_2_. Fresh aAPCs were added weekly to maintain stimulation. Cells were cultured in complete RPMI‐1640 medium supplemented with 300 IU/mL IL‐2 at a density not exceeding 1 × 10^6^ cells/mL. The proportion of induced NK cells was verified by flow cytometry.

### 4.20 γδ T Cell Expansion

PBMCs were initially seeded at 4 × 10^6^ cells/mL in γδ T cell complete medium (Cat# GMP‐CM3102A, Acro) containing 10% human AB serum (Cat# 100‐512‐100, GemCell), 7 µM zoledronic acid (Cat# HY‐13777, MCE), and 100 IU/mL IL‐2 and cultured at 37°C in a humidified incubator with 5% CO_2_. During subsequent culture, fresh complete medium containing 10% human AB serum and 100 IU/mL IL‐2 was added as needed to maintain a cell density of approximately 5 × 10^5^ cells/mL. The proportion of expanded γδ T cells was verified by flow cytometry.

### 4.21 Statistical Analysis

Quantitative data are presented as mean ± SEM, with individual data points shown. Where applicable, data were normalized according to the requirements of the corresponding experimental assays, as described in the relevant Methods sections. Sample sizes are reported in the corresponding figure legends and refer to biological replicates, technical replicates, independent donors, or mice, as appropriate for each experiment. Data satisfied the assumptions of normality (Shapiro–Wilk test) and homogeneity of variance (Levene's test), unless otherwise indicated. Two‐group comparisons were performed using unpaired two‐tailed Student's *t*‐tests. One‐way ANOVA with Tukey's post hoc test was used for single‐factor comparisons, and two‐way repeated‐measures ANOVA with Tukey's multiple‐comparisons test was used for longitudinal data. Survival curves were analyzed by the Kaplan–Meier method and compared using the log‐rank test. Statistical outliers were identified using the ROUT method (Q = 1%) and excluded when detected, with annotation in the corresponding figure legends. Statistical significance was defined as *p* < 0.05. *p* values are reported as not significant (ns), *p < 0.05, **p < 0.01, ***p < 0.001, or ****p < 0.0001. All analyses were performed using GraphPad Prism software V8.0. Figure assembly, schematic diagrams, and other graphical elements were prepared using Adobe Illustrator 2020 (V 24.0.1).

## Author Contributions

W.X., L.Y., and A.H.C. conceived the study and supervised the research. L.W., Y.Z., S.P., and C.H. performed the experiments and investigation; C.H. performed validation. H.G. and T.Z. developed the methodology. L.W. prepared the original draft. L.Y. and A.H.C. reviewed and edited the manuscript. W.X. and L.Y. acquired funding. All authors discussed the results and approved the final manuscript.

## Ethics Statement

Human peripheral blood mononuclear cells used for CAR‐T cell generation were obtained from healthy donors through YAYU Bio. Blood was collected after written informed consent was obtained from all donors and with approval from Shanghai Liquan Hospital (Approval No. SHLQ‐YLK‐2022‐09). Buffy coats were de‐identified prior to our receipt, and were therefore used anonymously. Donor demographic information was provided by YAYU Bio but was not used for analyses. All animal experiments were conducted in compliance with the ARRIVE guidelines and were approved by the Institutional Animal Care and Use Committee of Shanghai Ninth People's Hospital (Approval No. SH9H‐2024‐A1276‐1).

## Conflicts of Interest

A.H.C. is a founding member of Shanghai YaKe Biotechnology Ltd., a biotechnology company focused on the research and development of cellular immunotherapies for cancer. Y.Z. and S.P. are employees of Shanghai YaKe Biotechnology Ltd. A.H.C., Y.Z., and S.P. are named on a patent application related to this work. The other authors declare no competing interests.

## Supporting information




**Supporting File 1**: advs77992‐sup‐0001‐SuppMat.docx.

**Supporting File 2**: advs77992‐sup‐0002‐SuppMat.xlsx.

## Data Availability

The data that support the findings of this study are openly available in GEO database at https://www.ncbi.nlm.nih.gov/geo/query/acc.cgi?acc = GSE302681, reference number GSE302681.
