# Supplementary material for: A Multi‐Targeting Chimeric Antigen Receptor‐T Cell Platform to Overcome Antigen Heterogeneity in the Treatment of Non‐Small Cell Lung Cancer
Source: Adv Sci (Weinh). 2026 Sep 27:e77992. Online ahead of print. doi: 10.1002/advs.77992 (PMC13616289; doi:10.1002/advs.77992)
Supplement: Supplementary file 1 [file ADVS-9999-e77992-s001.docx]

Supporting Information

**A Multi-targeting Chimeric Antigen Receptor-T Cell Platform to Overcome Antigen Heterogeneity in the Treatment of Non-small Cell Lung Cancer**

*Lina Wang, Yanlei Zhang, Shuixiu Peng, Chenchen Hou, Huaqi Guo, Tianyu Zhou, Alex H. Chang*, Lifeng Yan*, and Weining Xiong**

**Supplementary Figures**

**Figure S1**

**Figure S1.** **CD276 and NKG2DLs are highly expressed in NSCLC**

**(A)** Box plots showing mRNA expression of eight NKG2DLs in human normal (n = 110) and NSCLC (n = 1016) samples based on TCGA. The whiskers indicate minimum to maximum values. An unpaired, two-tailed Student’s *t* test was used to calculate the significant difference between 2 groups. ns, not significant. *****P* < 0.0001.

**(B)** Detection of CD276 and NKG2DLs expression by flow cytometry in human NSCLC cell lines.

**Figure S2**

**Figure S2. Supplementary validation of CAR expression**

**(A** and **B)** Transduction efficiency and stability of CAR-T cells on day 10 (**A**) and day 14 (**B**) after lentiviral transduction, as assessed by DeepTag staining. The flow cytometry histograms in (A) and (B) show the same batch of CAR-T cells as in Figure 2B. Data are presented as mean ± SEM of untransduced T (UTD) or CAR-T cells generated from three independent donors.

**(C)** CAR expression in the same batch of CAR-T cells as shown in Figure 2B, assessed by anti-NKG2D monoclonal antibody staining. Corresponding quantification data are presented as mean ± SEM of CAR-T cells generated from three independent donors.

**Figure S3**

**Figure S3.** **Reversing the spatial order of CD276 antigen recognition domain (ARD) and NKG2D extracellular domain (ECD) does not improve CAR-T cell expression or antitumor function**

**(A)** Representative flow cytometry histograms showing CAR expression detected by DeepTag in untransduced T (UTD), CD276-NKG2DLs BB002 CAR, NKG2DLs-CD276 BB002 CAR, and CD276-NKG2DLs Bicephali CAR T cells. Data shown are from one of three independent donors.

**(B-D)** Real-time cell analysis (RTCA) showing cytotoxic activity of UTD and CAR-T cells against A549 (**B**), A549-CD276 knockout (**C**), and LLC-CD276⁺ (**D**) tumor cells. Effector-to-target (E:T) cell ratio = 1:1. Representative cytotoxicity data from three independent experiments using T cells from three healthy donors are shown.

**(E-G)** Specific lytic ability of CAR-T cells against A549 (**E**), A549-CD276 knockout (**F**), and LLC-CD276⁺ (**G**) tumor cells obtained from the RTCA analysis. Data are presented as mean ± SEM of three replicates. Comparisons among groups were conducted by unpaired, two-tailed Student’s *t* test. **P* < 0.05 and ***P* < 0.01.

**(H)** Representative flow cytometry histograms showing CAR expression detected by anti-NKG2D monoclonal antibody in NKG2DLs BB002 CAR and NKG2DLs Bicephali CAR-T cells. The NKG2D ECD was displayed N-terminally in the NKG2DLs-BB002 CAR and C-terminally in the NKG2DLs-Bicephali CAR. Data shown are from one of three independent donors.

**(I)** RTCA analysis showing cytotoxic activity of UTD and CAR-T cells against A549 tumor cells. Effector-to-target (E:T) cell ratio = 1:1. Representative cytotoxicity data from three independent experiments using T cells from three healthy donors are shown.

**(J)** Specific lytic ability of CAR-T cells against A549 tumor cells obtained from the RTCA analysis. Data are presented as mean ± SEM of three replicates. Comparison among groups was conducted by unpaired, two-tailed Student’s *t* test. ***P* < 0.01.

**Figure S4**

**Figure S4. Bicephali CAR-T cells exhibit enhanced cytotoxicity, effector cytokine secretion, and antigen-dependent proliferation across multiple NSCLC models**

**(A** and **B)** xCELLigence RTCA was used to monitor the cytolysis of CD276 and NKG2DLs multi-targeting CAR-T cells against the human NSCLC tumor cell lines H1299 (**A**) and PC9 (**B**). Effector-to-target (E:T) cell ratio = 1:1. Representative cytotoxicity data from three independent experiments using T cells from three healthy donors are shown.

**(C-G)** Analysis of the levels of cytokines IL-2 (**C**), IFN-γ (**D**), TNF-α (**E**), granzyme B (**F**), and perforin (**G**) in the supernatants of untransduced T (UTD) or CAR-T cells co-cultured with A549 cells for 24 hours. E:T ratio=1:1. Data are presented as mean ± SEM of three replicates. Comparisons among groups were conducted by unpaired, two-tailed Student’s *t* test. **P* < 0.05 and ***P* < 0.01.

**(H-J)** CFSE-labeled UTD and CAR-T cells were co-cultured with A549 (**H**), H1299 (**I**), or PC9 (**J**) tumor cells for 3 days, and antigen-dependent proliferation was analyzed by flow cytometry. Dotted lines indicate initial CFSE fluorescence intensity, and solid lines indicate CFSE fluorescence intensity after 3 days of co-culture, reflecting CFSE dilution associated with cell proliferation. Representative data from one of three donors are displayed.

**Figure S5**

**Figure S5. CD276/NKG2DLs Bicephali CAR-T cells formed stable immunological synapse (IS) than BB002 CAR-T cells**

**(A)** Representative confocal microscopy images of synapses formed by BB002 or Bicephali CAR-T cells in conjugation with A549 cells. For each group, 20 random cells were imaged and evaluated. F-actin (red), CAR-T cells (green), anti-Pericentrin-labeled microtubule organizing center (MTOC, magenta), and nuclei (DAPI, blue) are shown. Scale bar = 2.5μm.

**(B)** Quantification of IS area (μm^2^) formed by BB002 and Bicephali CAR-T cells upon target engagement. For each group, 20 random cells were imaged and evaluated. Data are presented as mean ± SEM. Statistical significance was determined by unpaired, two-tailed Student’s *t* test. ***P* < 0.01.

**(C)** Mean fluorescence intensity (MFI) of F-actin accumulation at the immune synapse interface in BB002 and Bicephali CAR-T cells. For each group, 20 random cells were imaged and evaluated. Data are presented as mean ± SEM. Statistical significance was determined by unpaired, two-tailed Student’s *t* test. ***P* < 0.01.

**(D)** Distance between MTOC and IS (μm) in BB002 and Bicephali CAR-T cells, indicating the extent of cellular polarization. For each group, 20 random cells were imaged and evaluated. Data are presented as mean ± SEM. Statistical significance was determined by unpaired, two-tailed Student’s *t* test. ***P* < 0.01.

**Figure S6**

**Figure S6. CD276/NKG2DLs Bicephali CAR-T cells exhibit enhanced in vivo persistence in tumor-bearing mice**

**(A)** Representative flow cytometry plots showing circulating human T cells (CD45⁺) in peripheral blood of tumor-bearing mice on days 21 and 28 after CAR-T cell infusion.

**(B)** Quantification of circulating human T cells per μL of peripheral blood at days 21 and 28 post-infusion, as determined by flow cytometry using absolute counting beads.

**Figure S7**

**Figure S7.** **Cytotoxicity of CAR-T cells against CD276/NKG2DLs co-expressing NSCLC cells**

**(A** and **B)** xCELLigence RTCA was used to monitor the cytolysis of human NSCLC tumor cell lines H1299 (**A**) and PC9 (**B**) by CAR-T cells. Effector-to-target (E:T) cell ratio = 1:1. Representative cytotoxicity data from three independent experiments using T cells from three healthy donors are shown.

**(C** and **D)** Specific lytic ability of CAR-T cells against the human NSCLC tumor cell lines H1299 (**C**) and PC9 (**D**) at various E:T ratios obtained from the RTCA analysis. Data are presented as mean ± SEM of three replicates. Comparisons among groups were conducted by two-way ANOVA with Tukey’s multiple comparisons test. *****P* < 0.0001.

**(E**-**I)** Analysis of the levels of the cytokines IL-2 (**E**), IFN-γ (**F**), TNF-α (**G**), granzyme B (**H**), and perforin (**I**) in the supernatants of untransduced T (UTD) or CAR-T cells co-cultured with H1299 or PC9 cells for 24 hours. E:T ratio=1:1. Data are presented as mean ± SEM of three replicates. Comparisons among groups were conducted by unpaired, two-tailed Student’s *t* test. **P* < 0.05 and ***P* < 0.01. ns, not significant.

**Figure S8**

**Figure S8. The body weight and in vivo safety evaluation of mice treated with UTD and CAR-T cells**

**(A)** Body weights of the mice treated with UTD and CAR-T cells described in Figure 5H. Data are presented as mean ± SEM (n = 5).

**(B)** Several vital organs including the brain, heart, liver, spleen and kidney were collected from mice bearing A549-GFPluc metastatic xenografts treated with UTD or CAR-T cells (n = 5). Formalin-fixed paraffin-embedded tissue sections were prepared for HE staining to assess any pathological damage. Scale bars = 100 μm.

**Figure S9**

**Figure S9.** **CD276 and NKG2DLs expression in A549-CD276 KO and LLC-CD276^+^ cell lines was assessed by flow cytometry**

**(A)** Flow cytometry plots and mean fluorescence intensity (MFI) showing the expression of CD276 in A549 wild-type cells and A549 cells with CD276 knockout (A549-CD276 KO) by CRISPR-Cas9.

**(B)** Murine Lewis lung carcinoma (LLC) cells were engineered to express human CD276. Flow cytometry plots and MFI showing the expression of CD276 in wild-type LLC cells and LLC cells transduced with a lentiviral vector encoding human CD276 (LLC-CD276^+^).

**(C)** Detection of NKG2DLs expression by flow cytometry in LLC-CD276^+^ tumor cells.

**Figure S10**

**Figure S10.** **NKG2DLs-targeting CAR-T cells preserve NK cell effector function and do not impair NK-mediated anti-tumor activity in vitro**

**(A)** Representative flow cytometry plots showing successful induction and identity (CD3⁻CD56⁺) of NK cells. Data shown are from one of three independent donors.

**(B)** Representative histograms of CAR expression in untransduced (UTD), CD276 CAR, NKG2DLs CAR, and Bicephali CAR-T cells. Data shown are from one of three independent donors. The percentage of CAR-positive cells is indicated.

**(C** and **D)** Representative flow cytometry plots (**C**) and quantification (**D**) of NK cell degranulation (CD56⁺CD107a⁺) after co-culture with A549 NSCLC cells and UTD, CD276 CAR, NKG2DLs CAR, or Bicephali CAR-T cells. Data are presented as mean ± SEM of NK cells generated from three independent donors. Statistical significance was determined by unpaired, two-tailed Student’s *t* test. ns, not significant.

**(E** and **F)** Representative flow cytometry plots (**E**) and quantification (**F**) of IFN-γ production in NK cells (CD56⁺IFN-γ⁺) after co-culture with A549 NSCLC cells and UTD, CD276 CAR, NKG2DLs CAR, or Bicephali CAR-T cells. Data are presented as mean ± SEM of NK cells generated from three independent donors. Statistical significance was determined by unpaired, two-tailed Student’s *t* test. ns, not significant.

**(G**-**J)** Effect of CAR-T cells on NK cell-mediated cytotoxicity. A549 (**G** and **H**) or H1299 (**I** and **J**) target cells were co-cultured with UTD or CAR-T cells alone (**G** and **I**), or in combination with NK cells (**H** and **J**). Cytotoxicity was monitored using the xCELLigence RTCA system. Effector-to-target (E:T) cell ratio = 1:1. Representative data from three independent experiments using PBMCs from three healthy donors are shown.

**(K**-**N)** Effect of irradiated CAR-T cells on NK cell-mediated cytotoxicity. A549 (**K** and **L**) or H1299 (**M** and **N**) target cells were co-cultured with irradiated UTD or CAR-T cells alone (**K** and **M**), or in combination with NK cells (**L** and **N**). Cytotoxicity was monitored using the xCELLigence RTCA system. Effector-to-target (E:T) cell ratio = 1:1. Representative data from three independent experiments using PBMCs from three healthy donors are shown.

**Figure S11**

**Figure S11.** **NKG2DLs-targeting CAR-T cells preserve γδ T cell effector function and do not impair γδ T-mediated anti-tumor activity in vitro**

**(A)** Representative flow cytometry plots showing successful induction and identity (CD3^+^TCR-γδ⁺) of γδ T cells. Data shown are from one of three independent donors.

**(B)** Representative histograms of CAR expression in untransduced (UTD), CD276 CAR, NKG2DLs CAR, and Bicephali CAR-T cells. Data shown are from one of three independent donors. The percentage of CAR-positive cells is indicated.

**(C** and **D)** Representative flow cytometry plots (**C**) and quantification (**D**) of γδ T cell degranulation (TCR-γδ⁺CD107a⁺) after co-culture with NSCLC cell line A549 and UTD, CD276 CAR, NKG2DLs CAR, or Bicephali CAR-T cells. Data are presented as mean ± SEM of γδ T cells generated from three independent donors. Statistical significance was determined by unpaired, two-tailed Student’s *t* test. ns, not significant.

**(E** and **F)** Representative flow cytometry plots (**E**) and quantification (**F**) of IFN-γ production in γδ T cells (TCR-γδ⁺IFN-γ⁺) after co-culture with NSCLC cell line A549 and UTD, CD276 CAR, NKG2DLs CAR, or Bicephali CAR-T cells. Data are presented as mean ± SEM of γδ T cells generated from three independent donors. Statistical significance was determined by unpaired, two-tailed Student’s *t* test. ns, not significant.

**(G**-**J)** Effect of CAR-T cells on γδ T cell-mediated cytotoxicity. A549 (**G** and **H**) or H1299 (**I** and **J**) target cells were co-cultured with UTD or CAR-T cells alone (**G** and **I**), or in combination with γδ T cells (**H** and **J**). Cytotoxicity was monitored using the xCELLigence RTCA system. Effector-to-target (E:T) cell ratio = 1:1. Representative data from three independent experiments using PBMCs from three healthy donors are shown.

**(K**-**N)** Effect of irradiated CAR-T cells on γδ T cell-mediated cytotoxicity. A549 (**K** and **L**) or H1299 (**M** and **N**) target cells were co-cultured with irradiated UTD or CAR-T cells alone (**K** and **M**), or in combination with γδ T cells (**L** and **N**). Cytotoxicity was monitored using the xCELLigence RTCA system. Effector-to-target (E:T) cell ratio = 1:1. Representative data from three independent experiments using PBMCs from three healthy donors are shown.

**Figure S12**

**Figure S12.** **Transcriptomic profiling of CAR-T cells versus untransduced T cells following 24 hours of coculture with A549 tumor cells**

**(A**-**C)** Heatmaps displaying differentially expressed genes (DEGs) between distinct CAR-T cells and UTD (untransduced T cells).

**(D**-**F)** Bubble plots showing the results of the KEGG enrichment analysis of upregulated DEGs in CAR-T cells relative to those in UTD.
